# Advances in the Application of Perovskite Materials

**DOI:** 10.1007/s40820-023-01140-3

**Published:** 2023-07-10

**Authors:** Lixiu Zhang, Luyao Mei, Kaiyang Wang, Yinhua Lv, Shuai Zhang, Yaxiao Lian, Xiaoke Liu, Zhiwei Ma, Guanjun Xiao, Qiang Liu, Shuaibo Zhai, Shengli Zhang, Gengling Liu, Ligang Yuan, Bingbing Guo, Ziming Chen, Keyu Wei, Aqiang Liu, Shizhong Yue, Guangda Niu, Xiyan Pan, Jie Sun, Yong Hua, Wu-Qiang Wu, Dawei Di, Baodan Zhao, Jianjun Tian, Zhijie Wang, Yang Yang, Liang Chu, Mingjian Yuan, Haibo Zeng, Hin-Lap Yip, Keyou Yan, Wentao Xu, Lu Zhu, Wenhua Zhang, Guichuan Xing, Feng Gao, Liming Ding

**Affiliations:** 1 Center for Excellence in Nanoscience (CAS), Key Laboratory of Nanosystem and Hierarchical Fabrication (CAS), National Center for Nanoscience and Technology, Beijing, 100190 People’s Republic of China; 2 University of Chinese Academy of Sciences, Beijing, 100049 People’s Republic of China; 3 Department of Physics, Linköping University, 58183 Linköping, Sweden; 4 Institute of Applied Physics and Materials Engineering, University of Macau, Macau, 999078 People’s Republic of China; 5 School of Materials Science and Engineering, Yunnan University, Kunming, 650091 People’s Republic of China; 6 School of Microelectronics Science and Technology, Sun Yat-sen University, Zhuhai, 519082 People’s Republic of China; 7 College of Electronic Information and Optical Engineering, Nankai University, Tianjin, 300350 People’s Republic of China; 8 Guangdong Provincial Key Laboratory of Semiconductor Optoelectronic Materials and Intelligent Photonic Systems, Harbin Institute of Technology, Shenzhen, 518055 People’s Republic of China; 9 School of Materials Science and Engineering, Nanjing University of Science and Technology, Nanjing, 210094 People’s Republic of China; 10 College of Optical Science and Engineering, Zhejiang University, Hangzhou, 310027 People’s Republic of China; 11 State Key Laboratory of Superhard Materials, Jilin University, Changchun, 130012 People’s Republic of China; 12 College of Electronic and Optical Engineering, Nanjing University of Posts and Telecommunications, Nanjing, 210023 People’s Republic of China; 13 School of Chemistry, Sun Yat-sen University, Guangzhou, 510006 People’s Republic of China; 14 School of Environment and Energy, South China University of Technology, Guangzhou, 510000 People’s Republic of China; 15 Department of Chemistry, Imperial College London, London, W12 0BZ UK; 16 College of Chemistry, Nankai University, Tianjin, 300071 People’s Republic of China; 17 Institute for Advanced Materials and Technology, University of Science and Technology Beijing, Beijing, 100083 People’s Republic of China; 18 Institute of Semiconductors, Chinese Academy of Sciences, Beijing, 100083 People’s Republic of China; 19 School of Optical and Electronic Information, Huazhong University of Science and Technology, Wuhan, 430074 People’s Republic of China; 20 School of Electronics and Information, Hangzhou Dianzi University, Hangzhou, 310018 People’s Republic of China; 21 Department of Materials Science and Engineering, City University of Hong Kong, Hong Kong, 999077 People’s Republic of China

**Keywords:** Perovskites, Optoelectronic devices, Neuromorphic devices, Pressure-induced emission

## Abstract

Nowadays, the soar of photovoltaic performance of perovskite solar cells has set off a fever in the study of metal halide perovskite materials. The excellent optoelectronic properties and defect tolerance feature allow metal halide perovskite to be employed in a wide variety of applications. This article provides a holistic review over the current progress and future prospects of metal halide perovskite materials in representative promising applications, including traditional optoelectronic devices (solar cells, light-emitting diodes, photodetectors, lasers), and cutting-edge technologies in terms of neuromorphic devices (artificial synapses and memristors) and pressure-induced emission. This review highlights the fundamentals, the current progress and the remaining challenges for each application, aiming to provide a comprehensive overview of the development status and a navigation of future research for metal halide perovskite materials and devices.

## Highlights

- A comprehensive summary of the representative promising applications of metal halide perovskite materials, including traditional optoelectronic devices (solar cells, light-emitting diodes, photodetectors, lasers), and cutting-edge technologies in terms of neuromorphic devices (artificial synapses and memristors) and pressure-induced emission.
- For each application, the fundamentals of the field, the current progress and the remaining challenges are provided, based on the up-to-date works.

## Graphical abstract

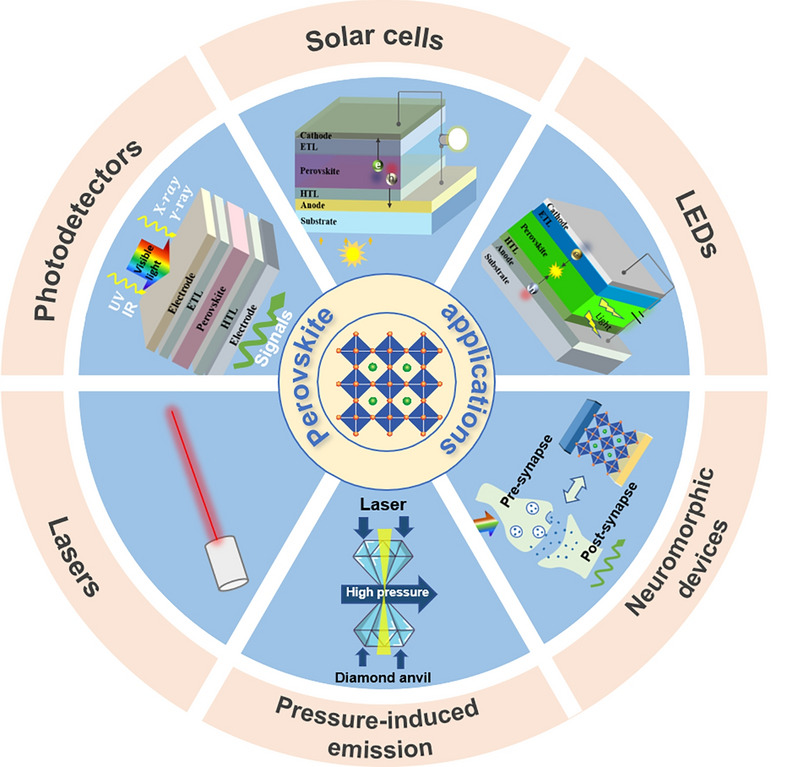

## Introduction

In recent years, metal halide perovskite (MHP) has demonstrated its exceptional capabilities in the optoelectronic field, which can be ascribed to its outstanding intrinsic photoelectric properties, such as high light harvesting ability, long and balanced carrier diffusion length, high defect tolerance, high photoluminescence quantum yield and readily tunable bandgap. Solution processible, flexible and cost-effective features make perovskite materials even more appealing to industry community. After decades of investigating, perovskite has been employed as the active material in various fields, including solar cells [1–3], light-emitting diodes (LEDs) [4–7], photodetectors [8–12], lasers [13, 14], memristors [15], artificial synapses devices [16, 17], pressure-induced emission [18, 19] and so on [20].

Perovskite, however, is not a novel material, the study of which could date back to 1839 when the first calcium titanate compounds (CaTiO_3_) mineral was discovered. It was named “perovskite” to commemorate the Russian mineralogist Lev Perovski, designating materials with the same crystal structure as CaTiO_3_. Today, the term “perovskite” in optoelectronic community usually refers to metal halide perovskite with the formula of ABX_3_, where A stands for monovalent cations like CH_3_NH_3_^+^ (MA^+^), CH(NH_2_)_2_^+^ (FA^+^) and Cs^+^; B for divalent metal cations like Pb^2+^ and Sn^2+^; and X for halide ions: I^−^, Br^−^ and Cl^−^. In this structure, the larger A^+^ cation coordinates with twelve X^−^ anions, occupying a cubo-octahedral void, while the smaller B^2+^ cation coordinates with six X^−^ anions, occupying an octahedral void.

Lead halide perovskite has been studied for a long history. Wells first synthesized lead halide perovskite in 1892 [21], which did not attract much attention until M. Era et al. investigated the electronic properties of 2D layered halide perovskite and applied it to electroluminescent device in 1994 [22]. In 1999, Kagan et al. used organic–inorganic hybrid perovskite (OIHP) as the semiconducting channels in field effect transistors [23]. In 2009, Miyasaka et al. firstly utilized methyl ammonium lead iodide (MAPbI_3_) as the sensitizer in dye-sensitized solar cell and achieved a power conversion efficiency (PCE) of 3.8% [24]. Based on this pioneering work, numerous studies on perovskite solar cells (PSCs) have sprung up. Nowadays, the champion efficiency of PSCs has reached 26.0% [25], motivating the attempt for stepping into commercialization. In addition to PSCs, other perovskite-based optoelectronic devices are also flourishing in these years. For perovskite LEDs, the idea of utilizing perovskites as electroluminescent materials was earlier than PSCs but the actual progress started from 2014, when Friend et al. prepared MAPbX_3_ thin-film-based perovskite LEDs with external quantum efficiency (EQE) less than 1% [26]. After around ten years of developing, the EQE of green, red and near-infrared (NIR) LEDs have all surpassed 20% [4–6], with the current highest EQE reaching up to 28.9% for green perovskite LEDs [7]. Due to the high color purity and luminescence efficiencies, perovskite LEDs hold the promise of realizing full color display with a wide color gamut. At the same time period, perovskites have also demonstrated promising potential to be used as photodetector and imaging array materials, with extremely wide detective region ranging from UV–visible–NIR [8–10] to X-ray [11] and γ-ray detection [12]. Besides, owing to the amplified spontaneous emission (ASE) behavior of perovskite films, perovskite can achieve coherent light emission, making it capable to be used as lasing materials. After years of exploring, some figures of merit of perovskite lasers have been greatly improved [13, 14]. Furthermore, by virtue of the resistive switching ability of perovskite materials, novel electronic devices involving memristors [15] and artificial synapses [16, 17] are also investigated. In addition, pressure-induced emission [18, 19] have also been observed in perovskite materials, hastening the further applications in anti-counterfeiting, information storage, sensing and display.

The research community has made remarkable achievements in the fields of MHP materials and devices. There is a need to systematically survey the current status and progress for various applications of MHPS. In this review, we overview the fundamentals and current progress for different applications of perovskite materials, including the commonly studied optoelectronic devices (solar cells, LEDs, photodetectors and lasers), novel neuromorphic devices (memristors, artificial synapses) and pressure-induced emission. The key merits of each application based on up-to-date works are evaluated. Furthermore, insightful perspectives for remaining challenges and opportunities in each application are also provided, respectively.

## Perovskite Solar Cells

PSCs with perovskite as photoactive materials have achieved great progresses in efficiency, skyrocketing from 3.8% to 26.0% within only about a decade [25]. Since very rich literatures in this field offer numerous ways for a specific aspect, we will discuss the following topics to afford an overall understanding of the advancement of PSCs. First, the working mechanism will be introduced from the perspective of p–n junction, and several typical triiodide perovskite materials are elaborately summarized. Then, we describe the progress of device configuration innovation and some important reports in detail. Finally, we outline challenges and perspectives on the development of PSCs.

### Fundamentals

**Fig. 1:**
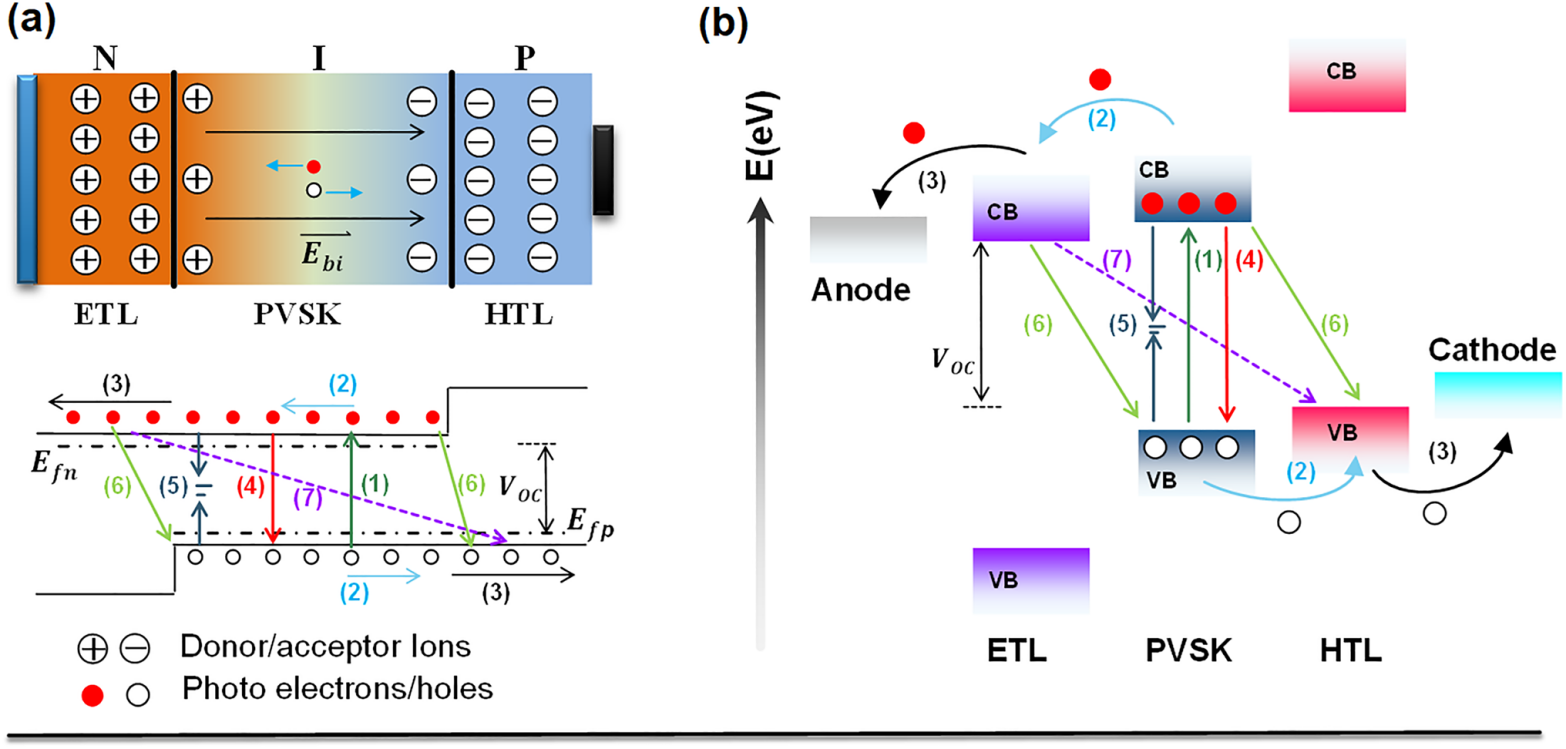
Schematic working principle in **a** P–I–N heterojunction and **b** perovskite solar cells

The PSC device can be regarded as a PIN heterojunction (Fig. 1a). The electron transport layer (ETL) and hole transport layer (HTL) serve as N-type and P-type semiconductors while perovskite is intrinsic layer (I), leading to the formation of built-in electric field (*E*_*bi*_). The photocarrier dynamics in PSCs include the following steps: charge generation, separation, transport, recombination and collection. In the charge generation process (1), perovskite films with narrower bandgaps possess wider absorption spectrum coverage, leading to higher usage of incident photons for photocurrent but at lower photovoltage. Exciton dissociation into free charge carriers must overcome the exciton binding energy (*E*_B_), which is easy for perovskite because the *E*_B_ is less than 50 meV [27].

Charge separation, including the charge drift and transfer toward N/P-type ETL/HTL (2), occurs after exciton dissociation, followed by charge extraction and transport (3) by ETL/HTL toward electrode (Fig. 1a). Charge radiative recombination (4) is inevitable, leading to photovoltage loss. Charge non-radiative recombination is always mediated by defects (5) and interface imperfection (6, 7). Charge diffusion length in the perovskite films is an important parameter to evaluate charge collection efficiency before recombination. Large grain size and low trap states can reduce the charge recombination in perovskite films and avoid the direct CTL contact to reduce the leakage current through process (5, 6, 7). The photoexcitation and recombination jointly determine the quasi-Fermi level difference, which reflects the open-circuit voltage (*V*_oc_, Fig. 1a).

In the charge collection step in PSCs, electrons and holes will be transferred to the corresponding electrodes. Generally, charge selective layers (ETL/HTL) are necessary at the interface between perovskite film and electrodes. These interfacial layers can reduce the energy barrier between perovskite film and electrode materials and suppress the potential interfacial charge recombination, especially the non-radiation recombination causing the large *V*_oc_ loss. Therefore, the photovoltage output is related to the energy difference of the conduction band (CB) of ETL and the valence band (VB) of HTL (Fig. 1b). Thus, interface engineering is critical to achieving effective charge collection.

### State-of-the-art Level

Based on the Shockley–Queisser (SQ limit) theory, the theoretical PCE (PCE^SQ limit^) for perovskite solar cells (1.53 eV) is 31.34%. After 14 years of studying, the PCE of current state-of-the-art PSC devices has been improved to 26.0% [25], which is 82% of its PCE^SQ limit^, comparable to benchmark GaAs and c-Si solar cells with 89.4% and 82.2% of their theoretical efficiencies, respectively (Table 1) [28]. Comparing the parameters, one can find PSCs still have a way to go to catch up with the performance of GaAs solar cells. The previous efficiency breakthrough can be mainly attributed to the growing understanding and precise control of the film crystallization process and device fabrication techniques. In order to go further, minimizing non-radiative recombination losses in perovskite layer, developing efficient charge extraction layers with the appropriate energy levels and reducing defects and surface states at the interface are of great significance.

**Table 1:** Record single‐junction solar cell under the global AM1.5 spectrum (1000 W m^−2^) at 25 °C [28]

	Bandgap (eV)	*V*_oc_ (V)	*J*_sc_ (mA cm^−2^)	FF (%)	PCE (%)	PCE^SQ limit^ (%)	PCE/PCE^SQ limit^ (%)
Perovskite	1.53	1.19	26.00	84	26.0	31.34	83.0
GaAs	1.43	1.1272	29.78	86.7	29.1	32.54	89.4
c–Si	1.11	0.738	42.65	84.9	26.7	32.55	82.2

### Typical Materials

In terms of dimensionality, perovskite can be divided into three types: three-dimensional (3D) perovskites, two-dimensional (2D) perovskites and zero-dimensional perovskites (quantum dots). In terms of chemical composition, perovskite materials can also be designated as organic–inorganic hybrid perovskite, all-inorganic perovskites and lead-free perovskites. These properties make perovskite crystallize in diversified ways, enriching the PSC material family. Here only some typical perovskite materials are presented, and detailed discussion can be found in the following sections.

*MAPbI*_*3*_ MAPbI_3_ perovskite exhibits excellent optoelectronic properties and can be easily processed, thus attracting great research interest in PSC study. However, limited by its fairly large bandgap (1.57 eV) [29], the PCE of MAPbI_3_-based cell is relatively low. Moreover, the thermal stability of MAPbI_3_ is poor because of the MA escape-induced device degradation. For instance, Park et al. reported that MA^+^ loss would become serious when aged temperature over 80 °C, leading to severe cell degradation [30]. Therefore, narrowing the band gap to the ideal band gap of ~ 1.34 eV (based on the SQ limit) for the perovskite is very essential to achieve the maximum PCE of PSCs.

*FAPbI*_*3*_ Density functional theory (DFT) calculations show FAPbI_3_ has a narrow bandgap of 1.43 eV [31], which lies in the ideal range to achieve very high efficiency of the single-junction solar cells. Moreover, FAPbI_3_ exhibits outstanding semiconducting properties, including a weaker electron–photon coupling, longer carrier lifetime and smaller charge carrier effective mass than MAPbI_3_. These remarkable properties make FAPbI_3_ very competitive for high-performance solar cells. However, the light active *α*-FAPbI_3_ is a metastable phase at room temperature and can easily transform into the insulating δ phase, which is a disaster for PSC device. Therefore, it is highly necessary to exploit strategies to stabilize the *α*-FAPbI_3_. Examples include composition tailoring, dimensionality engineering, substrate strain relaxation and crystallization regulation.

*CsPbI*_*3*_ All-inorganic PSCs exhibit excellent thermal stability compared with organic–inorganic PSCs with volatile organic components. The most extensively adopted all-inorganic perovskite material is cubic *α*-CsPbI_3_ because of its narrowest bandgap (1.73 eV), compared with those Br-containing species CsPb(I_1 − x_Br_x_)_3_. However, it is difficult to fabricate *α*-CsPbI_3_ at the ambient atmosphere due to the rapid phase transformation from *α*-CsPbI_3_ to the orthorhombic phase (*δ*-CsPbI_3_ with bandgap of 2.82 eV) [32]. Solution processing and vapor deposition methods are the two main techniques to fabricate *α*-CsPbI_3_ [33, 34]. Doping modulation [35], additive strategy [36], interface engineering [37] and dimensional regulation [38] are generally employed to improve the efficiency and stability of *α*-CsPbI_3_-based PSCs. For instance, Meng et al*.* utilized phenyltrimethylammonium iodide (PTAI) to fabricate low-dimensional (LD) perovskites on CsPbI_3_ surface, in which the LD perovskite not only enhances the phase stability of CsPbI_3_ but also effectively suppresses the non-radiative recombination, giving rise to a high efficiency of 21.0% with high stability [38].

### PSCs with Different Configurations

**Fig. 2:**
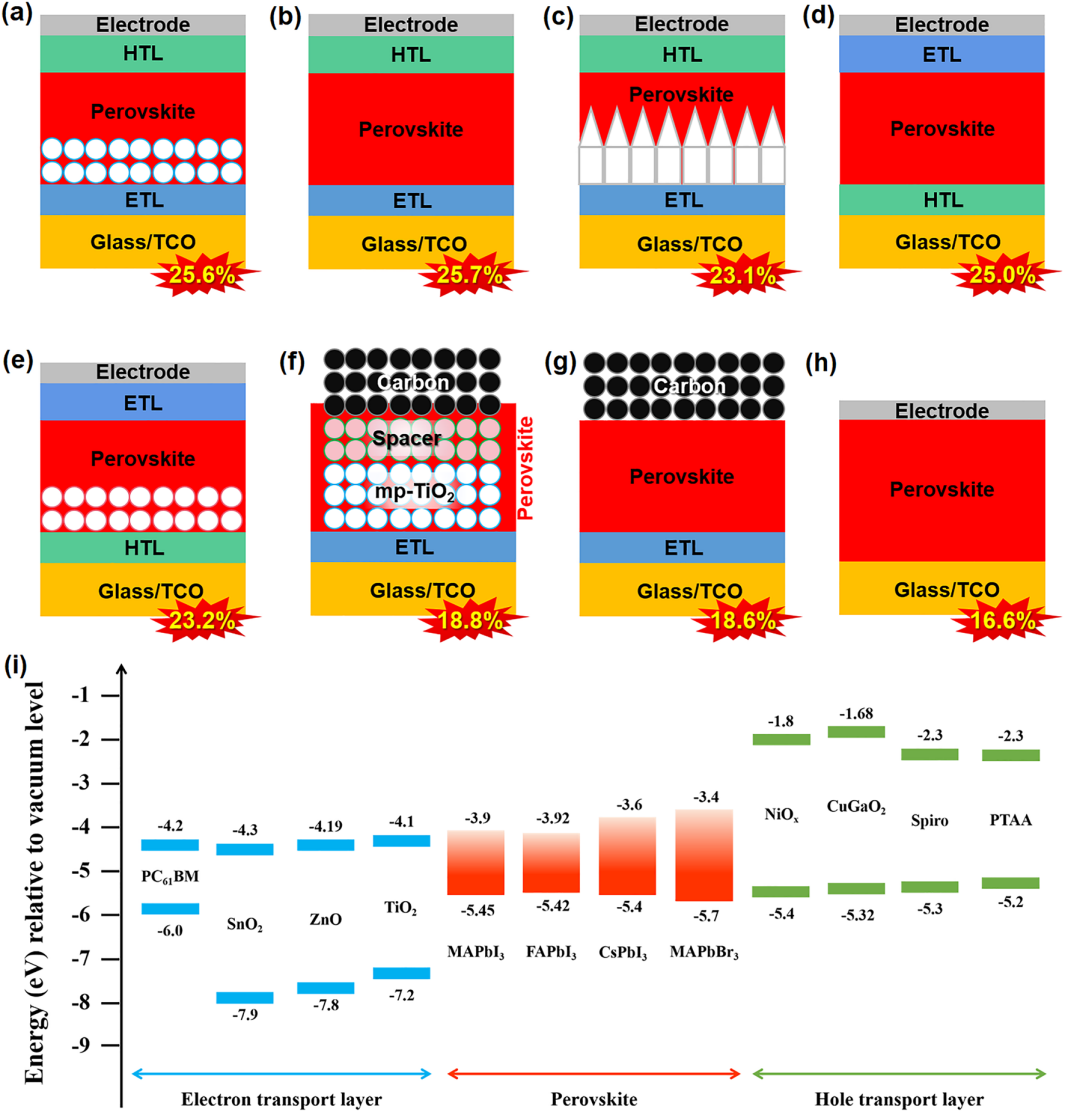
Schematic diagram of device architectures and energy level alignment. **a** Mesoscopic n–i–p, **b** planar n–i–p structure, **c** 3D n–i–p PSCs with 1D ETL, **d** planar p–i–n and **e** mesoscopic p–i–n PSCs. CTL-free PSCs: **f** HTL-free mesoscopic, **g** HTL-free planar and **h** HTL and ETL-free structure. **i** Energy band diagrams of charge transport materials and perovskite materials that are widely employed in PSCs [2, 39, 41–47]

**Fig. 3:**
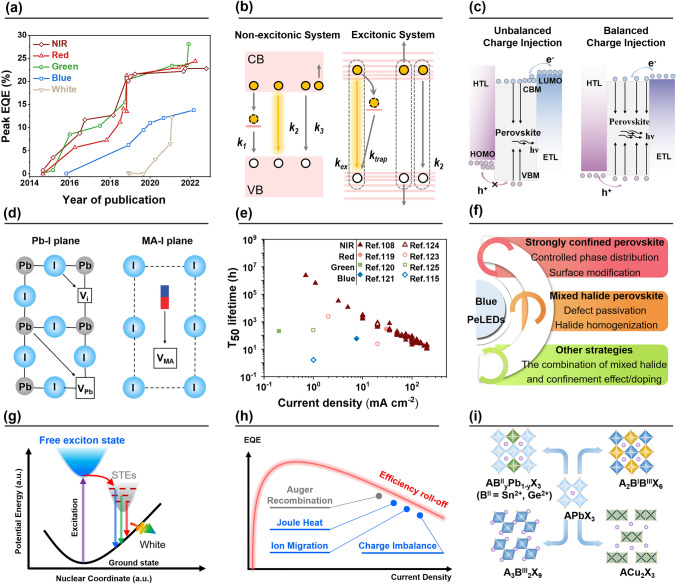
**a** Evolution of PeLEDs EQE over time [4–7, 26, 92–122]. **b** Non-excitonic and excitonic recombination models for perovskite emitters. **c** Charge injection and recombination in PeLEDs structures. **d** Ionic transport mechanisms in a CH_3_NH_3_PbI_3_ perovskite structure. Schematic illustration of the three ionic transport mechanisms involving conventional vacancy hopping between neighboring positions: I^−^ migration along an octahedron edge; Pb^2+^ migration along the diagonal direction < 110 > ; CH_3_NH_3_^+^ migration into a neighboring vacant A-site cage involving motion normal to the unit cell face composed of four iodide ions. **e** *T*_50_ lifetime data of some of the most stable PeLEDs with various colors from a selection of literature [108, 115, 119–121, 123–125]. **f** Strategies for improving the performance of blue PeLEDs. **g** Emission mechanisms of STEs. **h** Factors contributing to efficiency roll-off. **i** Crystal structures of lead-free perovskites

PSCs generally adopted layered configurations, consisting of a transparent conductive oxide (TCO) substrate, an n-type ETL, a perovskite light absorber layer, a p-type HTL and a counter electrode. According to the display order of functional layers, the structure of PSCs can be basically classified into n–i–p normal and p–i–n inverted and charge transport layer-free (CTL-free) configurations. Depending on the morphologies of the ETLs, the n–i–p-type PSCs can be further divided into mesoscopic structure with mesoporous ETLs (Fig. 2a), planar cell with compact thin-film ETLs (Fig. 2b) and 3D PSCs with one-dimensional (1D) ETLs (Fig. 2c). Similarly, the p–i–n inverted PSCs can be divided into two types: the planar configuration with compact thin-film HTL (Fig. 2d) and the mesoscopic structure with mesoporous HTLs (Fig. 2e). The difficulty in growing high-performance 1D HTMs hinders the study on the 3D inverted cells with 1D HTLs. Nowadays, both normal and inverted PSC devices have achieved over 25% PCE [3, 39, 40], which can be attributed to the matched energy level, reduced charge recombination and balanced carrier extraction enabled by appropriated charge transport layers (CTLs). However, the high-cost and complex fabrication process accompanied by the use of CTL limits the commercialization potential of PSCs. A simplified configuration called charge transport layer-free (CTL-free) structure (Fig. 2f–h) offers a feasible way to balance the efficiency and cost, with the PCE surpassing 21% [41]. For different device configurations, the energy level diagram alignment between CTL and perovskite layer should be carefully designed to facilitate charge carrier transport and extraction (Fig. 2i). The current progress of PSCs in each configuration is overviewed in the following section, and the detailed information is summarized in Table 2.

**Table 2:** Performances of the PSCs with varying architectures

Device configurations	Device structure	*V*_oc_ (V)	*J*_sc_ (mA cm^−2^)	FF (%)	PCE (%)	Stability test	References
Mesoscopic structure with mesoporous ETLs	FTO/c-TiO_2_/mp-TiO_2_/FAPbI_3_/octylammonium iodide/Spiro-OMeTAD/Au	1.19	26.35	82	25.6	RH ~ 20%, 1000 h; 60 °C, 1000 h; MPP, Xenon lamp, 12 h; MPP, LED, 450 h	[2]
Planar cell with compact thin-film ETLs	ITO/SnO_2_/FAPbI_3_/PEAI/Spiro-OMeTAD /Au	1.18	25.2	78	23.32	85 °C, 1000 h (PTAA as HTL)	[42]
	FTO/c-TiO_2_/paa-QD-SnO_2_/FAPbI_3_/octylammonium iodide/Spiro-OMeTAD/Au	1.176	26.09	84	25.71	Unsealed, 350 h MPP tracking test	[48]
	FTO/Cl-SnO_2_/FAPbI_3_/ MeO-PEAI/Spiro-OMeTAD/Au	1.189	25.71	84.43	25.83	> 90% of their initial PCE under 1 sun illumination for 500 h	[3]
	FTO)/SnO_2_/ FAPbI_3_/ MeO-PEAI/Spiro-OMeTAD/Au	1.179	25.80	84.60	25.73	~ 88% of the initial efficiency after 600 h with MPP tracking	[40]
3D PSCs with one-dimensional (1D) ETLs	FTO/c-TiO_2_/1D anatase TiO_2_ NPys/FAPbI_3_/ Spiro-OMeTAD/Au	1.17	24.06	78	22.48	85 °C, 1000 h (PTAA as HTL)	[43]
	ITO/c-TiO_2_/1D anataseTiO_2_ NRs/PMMA:PCBM/Cs_0.05_FA_0.88_MA_0.07_PbI_2.56_Br_0.44_/PMMA/P3HT:CuPc/Au	1.24	21.91	85	23.17	MPP tracking test; 85 °C, 85% RH	[49]
Planar configuration with compact thin-film HTL	FTO/NiMgLiO/ MAPbI_3_/ PCBM/Ti(Nb)O_x_/Ag	1.083	20.4	82.7	18.3	> 97% of their initial PCE in the dark for 1000 h	[50]
	ITO/*P*-PY/Cs_0.05_(FA_0.9_MA_0.1_)_0.95_Pb(I_0.9_Br_0.1_)_3_ / C_60_/BCP/Ag	1.15	23.75	82	22.40	40% RH, > 90% after 1000 h	[51]
	ITO/PTAA/Cl-PEAI/ Cs_0.05_(FA_5/6_MA_1/6_)_0.95_Pb(I_0.9_Br_0.1_)_3_/Cl-PEAI PCBM/BCP/Ag	1.15	24.29	83.55	23.34	None	[52]
	ITO/2PACz/FAMAPbI_3_ /2D perovskite/C60/BCP/Ag	1.21	24.31	82.6	24.30	> 95% of the initial PCE (T95) after > 1200 h	[53]
	ITO/PTAA/(FA_0.98_MA0.0_2_)_0.95_Pb(I_0.98_Br_0.02_)_3/_FcTc_2_/C_60_/BCP/Ag	1.18	25.68	82	25.0	MPP test; IEC61215:2016 Standards; 85% RH, 85 °C; − 40 °C (15-min dwell) to 85 °C (15-min dwell), ramp rate of 100 °C h^−1^	[39]
Mesoscopic structure with mesoporous HTLs	FTO/c-NiO_*x*_/mp-NiO_*x*_/Cs_0.05_(MA_0.15_FA_0.85_)_0.95_Pb(Br_0.15_I_0.85_)_3_/PCBM/BCP/Ag	1.09	23.8	79	20.2	Unsealed, RH 35 ± 5%, 25 °C 90%	[54]
	ITO/NiO_x_/CuGaO_2_/perovskite/ PCBM/BCP/Ag	1.13	22.23	79.96	20.13	> 90% of its initial efficiency after two months under ambient conditions	[55]
	FTO/NiO_x_/mp-M:CCO/perovskite/TiO_2_/PC_61_BM/BCP/Ag	1.14	24.01	79	21.64	Unsealed, 85 °C 81% for 1000 h	[56]
ETL-free PSC	ITO/MAPbI_3_-F4TCNQ/C_60_/BCP/Cu	1.10	22.70	81	20.2	RH ~ 20%, 25 °C ~ 92% for 500 h	[44]
	FTO/a-NbOH/Cs_0.07_FA_0.70_MA_0.23_PbI_3_/ Spiro-OMeTAD/Au	1.16	23.00	79	21.1	Continuous one sun illumination, N_2_ ~ 96% for 500 h	[41]
HTL-free PSCs	FTO/mp TiO_2_/mp-ZrO_2_/MAPbI_3_/Carbon	1.03	22.82	80	18.82	RH ~ 85%, 85 °C ~ 92.4% for 1340 h	[45]
	ITO/APTES-linked C_60_/ MAPbI_3_/carbon	1.12	22.72	73	18.64	RH ~ 30–50% ~ 96% for 3000 h	[46]
Both ETL and HTL-free PSCs	FTO/FAPbI_3_/MAPbI_3_ /Au	0.95	24.96	70	16.57	None	[47]

#### n–i–p (normal) Structure

##### Mesoscopic

Mesoscopic structure adopts mesoporous ETLs, which offer sufficient contact area with perovskite layer and facilitate the extraction of photogenerated electron. Simultaneously, the mesoporous ETL can act as the scaffold to regulate the crystal growth of perovskite films. TiO_2_ is the most commonly used mesoporous ETL material. Nazeeruddin et al*.* [57] recently synthesized single-crystalline TiO_2_ rhombohedral nanoparticles with exposed (001) facets to construct Rb_0.03_Cs_0.05_MA_0.05_FA_0.90_PbI_3_-based PSCs with a high efficiency of 24.05%. However, heteroatomic doping is essentially required to further enhance device performance by reducing the series resistance [58]. Additionally, TiO_2_ has a high photocatalytic activity upon ultraviolet (UV) illumination [59], exerting a fairly unfavorable effect on device stability. To tackle this dilemma, other metal oxides with low or non-photocatalytic activity, such as SnO_2_ [60], ZnO [61] and Zn_2_SnO_4_ [62] [63, 64], have been assessed as ETLs of PSCs. Among them, SnO_2_ exhibits high electron mobility (100–200 cm^2^ V^−1^ s^−1^) and UV resistance, achieving excellent PV performance and gaining much attention in recent years. Fang et al. developed a kind of bifunctional mesoporous SnO_2_ to improve carrier collection efficiency, enabling an impressive PCE of 22.40% for FA_1 − x_MA_x_I_3 − x_Br_x_-based PSCs [60]. It should be noted that pore-forming agents (e.g., terpineol and ethyl cellulose) are always introduced to obtain a mesoporous ETL. High-temperature sintering is necessary during the device fabrication, which is a high-energy-consumption process and restricts the substrate selection. Therefore, the planar PSCs with relatively simple structure have attracted increasingly research interest.

##### Planar

The ETLs in planar PSCs are usually thin compact films of oxides without pinholes, which shorten the travel length of the charge carriers and facilitate the immediate collection of extracted electrons by electrodes. High device performance can thus be obtained despite the relatively small ETL/perovskite contact area. In 2017, Sargent et al*.* reported a low-temperature synthesis of the chlorine-capped TiO_2_ colloidal nanocrystal film that leads to improved surface passivation and reduced interfacial recombination and a certified efficiencies of 20.1% for Cs_0.05_FA_0.81_MA_0.14_PbI_2.55_Br_0.45_ PSC was yielded [65]. Compared with TiO_2_, SnO_2_ is more suitable for planar PSCs [66, 67]. However, the low-temperature process of SnO_2_ would inevitably induce a large number of surface states; thus, a suitable surface passivation is favorable to yield a decent PCE for SnO_2_-based cells [48, 68, 69]. In situ encapsulation of SnO_2_ NCs by amorphous NbO_x_ with residue of Cl ligands can passivate the defects at the ETL/FA_1 − *x*_MA_*x*_PbI_3 − *y*_Cl_*y*_ perovskite interface, resulting in cell with a decent PCE of 24.0% [67]. Biguanide hydrochloride (BGCl) can be employed as a chemically linkers between the SnO_2_ ETL and perovskite through Lewis coordination/electrostatic coupling, reducing interfacial defects and yielding a certified PCE of 24.4% [69]. Seok et al. found that a crystalline FASnCl_x_ phase was formed between surface Cl-bonded SnO_2_ and perovskite film, which act as an atomically coherent interlayer to significantly reduce the interfacial charge recombination. This discovery verifies the feasibility for modification with Cl ligand in inorganic ETLs to passivate buried interface, enabling a record efficiency of 25.8% in planar PSCs [3].

##### PSCs with 1D ETLs

From the perspective of contact area between ETLs and perovskite in n–i–p structure, 1D oriented ETLs, such as nanowires (NWs) [70], nanotubes (NTs) [71], nanorod (NRs) [72, 73], nanocones (NCs) [74] and nanopyramids (NPys) [43], are the intermediates between the mesoscopic and planar ones, showing superior optical and electrical properties. Unfortunately, for a long time, PSCs with 1D building blocks always lag far behind those normal ones. It is until 2020 that a landmark cell efficiency approaching 22.5% was reported for PSCs with 1D ETLs, where single-crystalline anatase TiO_2_ NPys with varied lengths were designed and used as ETLs of PSCs [43]. The TiO_2_ NPys/perovskite heterostructures are featured with highly oriented electric field that can facilitate the charge collection of electrons/holes to the anode/cathode, respectively. More later, White et al. introduced a complex nanoimprint approach to fabricate highly ordered, 1D polycrystalline anatase TiO_2_ ETLs. Assisted by polymer passivation, the champion cell exhibited a record PCE of 23.17% for Cs_0.05_FA_0.88_MA_0.07_PbI_2.56_Br_0.44_-based PSCs with 1D ETLs [49].

#### p–i–n (inverted) Structure

##### Planar

Since the first report of inverted PSCs by Wen et al. [51], the inverted planar architecture has attracted increasing interest mainly owing to their potential in enhancing the robustness against cell stability. Up to date, a variety of conductive polymers, organic small molecules and inorganic semiconductors have been successfully adopted as HTLs in inverted planar cells (Fig. 2d). Poly(ethylenedioxythiophene):poly(styrene sulfonate) (PEDOT:PSS) is probably the most widely used HTL in inverted PSCs due to its relatively high intrinsic mobility and well-matched energy level with perovskite [75]. However, the acidic and hygroscopic properties of PEDOT:PSS can accelerate the perovskite degradation, significantly reducing device performance and stability. Poly(bis(4-phenyl) (2,4,6-trimethylphenyl) amine (PTAA) is a candidate for inverted PSCs due to its outstanding charge carrier mobility, chemical neutrality and optical transmittance [47, 76]. Moreover, its high hydrophobicity nature can enhance the device moisture stability [52]. Nevertheless, the high cost of PTAA limits their application in large-area modules. In contrast, organic small molecule-based HTLs exhibit a facile synthesis with much reduced cost and equally excellent cell performance. Wolf et al. have recently employed 2PACz as both HTL and surface modifying agent to fabricate device with the structural display of 2PACz/Cs_0.03_(FA_0.90_MA_0.10_)_0.97_PbI_3_/2D perovskite/C_60_/BCP/Ag. A maximum PCE of 24.3% was achieved, and the encapsulated device show superior stability, retaining of > 95% of its initial PCE aging at damp-heat test (85 °C and 85% relative humidity) for 1500 h [77].

Compared with the organic materials, inorganic p-type semiconductors have much better stability toward photodegrade, thermal aging and chemical etching; thus, they provide a high possibility to address the stability concerns for PSCs’ long-term deployment. NiO_x_-based HTLs were intensively studied in recent years. Han et al. developed a spray pyrolysis avenue to produce Li_0.05_Mg_0.15_Ni_0.8_O film, enabling a PCE > 15% and an excellent light soaking stability for PSCs [50]. However, the inverted PSCs with inorganic HTL was limited by their cell efficiency. Surface modification by organic materials is an efficient way to improve PCE [78].

##### Mesoscopic

Guo et al. [79] first proposed the NiO_x_-based mesoscopic PSCs (Fig. 2e), in which the mesoporous NiO_x_ (mp-NiO_x)_ film was prepared by a sol–gel method. Tress et al. employed a low-cost triblock copolymer template-assisted strategy to build the mp-NiO_x_ scaffold that effectively promotes the growth of the perovskite film with better surface coverage. Ultimately, a decent PCE of 20.2% for a typical composition of Cs_0.05_(MA_0.15_FA_0.85_)_0.95_Pb(Br_0.15_I_0.85_)_3_ was achieved for mp-NiO_x_-based inverted PSCs [54]. In 2018, Chen et al*.* exploited a bilayer structure to suppress charge recombination with cell architecture of FTO/NiO_x_/mesoporous CuGaO_2_/perovskite layer/ETL/Au. In such a cell, the HTL was made of the NiO_x_ compact thin film and the mesoporous CuGaO_2_, which show graded energy levels that favor the charge carrier transfer and collection. Accordingly, both high device performance and stability can be obtained simultaneously [55]. Similar design of the graded HTLs has been further extended to materials of Zn^2+^-doped CuGaO_2_ [80], Mg^2+^-doped CuCrO_2_ [56] and CuScO_2_, and both high efficiency (over 23%) and stability ( 82.8% was retained subject to thermal aging at 85 °C for 1200 h and ~ 90% upon light soaking for 1000 h) were again obtained.

As described above, charge transport layers (including HTLs and ETLs) play essential roles in achieving high-performance PSCs. Nevertheless, simple design of the cell structures is a strong drive force to understand the mechanism of perovskite solar cells and to further facilitate the commercialization of PSCs for the low-cost, large-scale fabrication. Charge transport layer-free (HTL-free) PSCs are especially promising in this issue.

#### CTL-free PSCs

The state-of-the-art high-performance PSCs heavily relied on the utilization of high-quality charge transport layers (CTLs), which require costly materials and tedious fabrication processes, thus impeding the low-cost implementation of PSCs. Perovskites enjoys ambipolar carrier transport characteristics, enabling themselves to be capable of transporting both electrons and holes. This, somewhat, diminishes the necessity of employing either ETL or the hole transport layer (HTL) for constructing a variety of PSCs with simplified structures (Fig. 2f–h). The concept of CTL-free PSCs brings an "out-of-the-box" approach for minimizing the fabrication cost while ensuring high device performance. Encouragingly, substantial breakthrough in efficiencies have been witnessed for all types of CTL-free devices, including ETL-free, HTL-free as well as both ETL and HTL-free PSCs, progressively narrowing the PCE gap between simplified PSCs and conventional CTLs-containing counterparts [25].

In recent few years, tremendous efforts, such as molecular doping, interfacial modification or additive/composition engineering, have been made on boosting the photovoltaic performances of CTL-free PSCs [81–84]. In 2018, Wu et al. demonstrated the molecular p-doping of MAPbI_3_ perovskites by 2,3,5,6-tetrafluoro-7,7,8,8-tetra-cyanoquinodimethane (F4TCNQ) to enhance the film conductivity and induce upward band bending at the ITO/perovskite, which greatly facilitated the interfacial hole extraction and collection even in the absence of HTL [44]. A champion efficiency of 20.2% has been achieved for the HTL-free PSCs made by blade coating method. ETL-free PSCs with Cs_0.07_FA_0.70_MA_0.23_PbI_3_ composition showed a PCE of over 21% by introducing an ultrathin amorphous niobium oxyhydroxide (a-NbOH) interlayer, which showcased multiple functions, namely, defect passivation, crystallization modulation, hole blocking and electron tunneling [41]. The combination of CTL-free structures and the carbon electrode seems to be an ideal option to balance the "efficiency–stability–cost" golden triangle [85]. Back to 2014, Han and coworkers innovatively developed a triple-stacked mesoporous architecture of HTL-free carbon-based PSCs (C-PSCs) composed of sequentially printed mesoporous TiO_2_, ZrO_2_ and carbon layers (Fig. 2f). Via composition engineering, crystallization modulation and defects passivation, the PCEs of these mesoporous HTL-free C-PSCs have elevated from 12.84% to 18.82% [45, 86–88]. The key to obtain high performance in such device is to cautiously modulate the permeation of perovskite ink into the thick mesoporous TiO_2_/ZrO_2_/Carbon scaffold, in which the perovskites crystallization and their contact with other functional materials are difficult to control. In view of this, Wu and coworkers developed a novel all-carbon-based HTL-free PSCs with planar structures (Fig. 2g) via full-solution blade coating, which is compatible to roll-to-roll manufacturing. With the assist of ultrathin 3-aminopropyl triethoxysilane (APTES) interfacial linker, the interfacial adhesion toughness between C_60_ ETL and ITO substrate can be greatly reinforced, which facilitated the interfacial charge transport and boosted the PCE up to 18.64% [46]. Very recently, to take full advantage of simplified device structure, Zhu and coworkers have developed the ultrasimplified devices in the absence of both ETL and HTL, and the relevant device achieved a PCE of 16.57%. The magic of this less burden device is the bilayer stacking of heterogeneous FAPbI_3_/MAPbI_3_ perovskites with tailored energy bands (Fig. 2h) [47].

Further PCE enhancement of CTL-free PSCs can be achieved by rational optimization of perovskite film quality (i.e., uniformity, crystallinity and surface coverage) and optoelectronic properties (i.e., conductivity, carrier diffusion lengths, built-in electric field, etc.), as well as the contact junctions and energy level alignment between perovskite layer and adjacent electrode. Though the performance is promising, the next big challenge is to fabricate the CTL-free PSCs in a large-scale manner, especially targeting to mini-modules or solar panels. In this case, the large interfacial series resistance at the TCO/perovskite interface in the absence of CTLs or other buffer layers should be overcome. In addition, the carrier dynamics and relevant device physics in CTL-free PSCs requires particular attention. Such a concept of ultrasimplified devices can be further extended to the other perovskite-based optoelectronic devices, such as LEDs, photodetectors and transistors.

### Challenges Ahead

In this section, we highlight the recent progress of PSCs in terms of device architecture. Thanks to the bipolar characteristics and extraordinary properties of perovskite materials, the device structure can be designed with very simple displays while superior PV performance can still be obtained. Although significant advancements have been made, some big challenges are remained to be tackled from science and applicable deployment points of views.

First, there is still a gap between the highest PCE and the SQ limit theory. Exploiting efficient charge extraction layers, manipulating perovskite film quality and developing novel interfacial layer are vital to minimize non-radiative recombination losses and further elevate the device performance. Second, the charge carrier kinetics and corresponding physical model should be illustrated to get a better understanding of the underlying physical process. Moreover, the relatively poor operational stability of PSCs is urgent to be addressed with respect to both the extrinsic and intrinsic factors, such as advanced encapsulation techniques, perovskite composition regulation and robust interface modification. Lastly, it is highly necessary to develop upscale fabrication and investigate the nucleation and crystallization process toward their industrial application.

We are optimistic about the future of PSCs, and we believe the large-scale deployment of the PV panels and energy storage device will be helpful for solving the environmental pollution issue and energy crisis.

## Perovskite LEDs

Perovskite light-emitting diodes (PeLEDs) [26] have emerged as a promising candidate for next-generation light sources owing to their excellent color purity, spectral tunability, high luminescence efficiencies and low processing costs. This section provided an overview of the emerging PeLEDs technology and highlighted recent progress from the perspectives of material design, emission mechanisms, device architectures, interfacial control and light outcoupling. The rapid and significant advances in PeLEDs indicate their bright future as next-generation light sources. Yet PeLEDs still face barriers hindering their commercialization. We have highlighted several key challenges including improving device stability, realizing high-performance blue and white PeLEDs, suppressing efficiency roll-off and reducing toxicity hazards. Possible solutions for overcoming these challenges have also been discussed to foster new breakthroughs. Besides, the achievements already made in PeLEDs and the unique properties of perovskites indicate that perovskite emitters could potentially go beyond the PeLEDs area; for instance, they show potential applications in electrically driven lasers [89], biomedical diagnosis [90] and spin LEDs [91].

### An Overview of the Field

Since the first report of room-temperature electroluminescence (EL) from halide perovskite in 2014 [26], the field has been advancing rapidly; the EQEs of PeLEDs exceeded the 20% milestone in 2018 [5, 92–94], followed by more recent works improving device EQEs to over 28% (Fig. 3a, Table 3) [4, 7].

**Table 3:** Summary of PeLEDs performance

Device structure	Emissive layer composition	λ_EL_ (nm)	EQE (%)	References
ITO/TiO_2_/perovskite/ F8/MoO_3_/Ag	MAPbI_3-x_Cl_x_ MAPbBr_3_	754 517	0.76 0.1	[26]
ITO/ZnO/PEIE/perovskite/ TFB/MoO_x_/Au	FAPbI_3_:5AVA	803	20.7	[92]
ITO/ZnO/PEIE/perovskite/ TFB/MoO_x_/Au	NMA-FAPbI_3_: poly-HEMA	800	20.1	[93]
ITO/TiO_2_/perovskite/ F8/MoO_x_/Au	(PEA)_2_(CH_3_NH_3_)_n−1_Pb_n_I_3n+1_	750	8.8	[96]
ITO/ZnO/PEIE/perovskite/ TFB/MoO_x_/Au	NMAFAPbI_y_Br_7-y_	763	11.7	[97]
ITO/ZnO/PEIE/perovskite/ TFB/MoO_x_/Au	FAPbI_3_: ODEA	800	21.6	[113]
ITO/ZnO/PEIE/perovskite/ TFB/MoO_x_/Au	FAPbI_3_: SFB10	803	22.8	[108]
ITO/PEDOT:PSS/ perovskite/B3PYMPM/LiF/Al	CsPbBr_3_ MABr	525	20.3	[94]
ITO/poly-TPD/LiF/ perovskite/TPBi/CsF/Al	PEABr:CsPbBr_3_: 18-crown-6: MPEG-MAA	514	28.1	[4]
ITO/SOCP/perovskite/ TPBi/LiF/Al	MAPbBr_3_:TPBi	540	8.53	[95]
ITO/NiO_x_/TFB/PVK/ perovskite/TPBi/LiF/Al	PBABr:PEABr:CsPbBr_3_:LiBr	518	23.6	[114]
ITO/PEDOT:PSS/poly-TPD/perovskite/ TPBi/LiF/Al	CsPbI_3_ QDs	653	21.3	[5]
ITO/ZnO/PEI/perovskite/ TCTA/MoO_3_/Au	CsPbI_3_ NCs:SrCl_2_	690	13.5	[105]
ITO/PEDOT:PSS/ perovskite/TPBi/LiF/Al	CsPbBr_3_:PEACl:2%YCl_3_:YCl_3_	490	11	[115]
ITO/m-PEDOT:PSS/ perovskite/TPBi/LiF/Al	Cs_x_EA_1−x_PbBr_3_:EABr	488	12.1	[99]
ITO/PEDOT:PSS:PFI/PVK/PEABr/ perovskite QDs/TPBi/LiF/Al	CsPb_1−x_Sr_x_Br_3_	495	13.8	[116]
ITO/PEDOT:PSS/TFB/ perovskite/TPBi/LiF/Al	CsPbI_3_	Wide spectrum	6.5	[110]
ITO/NiO_x_/PVK/perovskite/ TPBi/LiF/Al/Ag/LiF/perovskite NCs	PEABr:IPABr:CsBr:PbBr_2_ PBABr_1.4_(Cs_0.7_FA_0.3_PbBr_3_)	Wide spectrum	12.2	[111]

The unprecedented pace of development for PeLEDs can be attributed to the advances in materials development and device design. The functional layer materials and device architectures of early PeLEDs were directly inherited from that of perovskite solar cells and solution-processed OLEDs [26]. MHP show comparable optoelectronic properties to that of conventional III-V semiconductors and much simpler manufacturing processes [92, 95, 96]. The performance of PeLEDs could be improved by a range of approaches, including compositional engineering [96, 97], molecular additives/blends [92, 93, 98, 99] that passivate defects and improve morphology; and dimensionality control that tailors the energetic landscape and excited-state kinetics [93, 96, 97] of the emissive perovskites. Regardless of which emission mechanisms may be dominant for particular perovskite compositions, the key to improving the internal quantum efficiencies (IQEs) of EL toward unity lies in the ability of enhancing radiative recombination while suppressing non-radiative recombination losses [93].

Apart from the aforementioned aspects in materials development, the evolution of PeLEDs architectures played a key role in achieving the current state-of-the-art devices [5, 92, 96, 100–103]. A variety of device structures were carefully designed for PeLEDs with different colors, in consideration of issues such as charge injection, blocking and balance. Consensus has been reached that suppressing non-radiative recombination at the emissive layer/charge transport interfaces [15, 93, 104–106] of PeLEDs is equally important as managing recombination in the bulk. While the IQEs of PeLEDs have been shown to approach 100% [93], their EQEs are still limited by the relatively low optical outcoupling efficiency of the current device design [107], leaving much space for efficiency improvements through light outcoupling.

While we have witnessed great progresses in developing PeLEDs to become a commercially available technology, many challenges remain. The poor device stability has been considered the main obstacle toward practical applications. A very recent breakthrough in this area was the demonstration of ultrastable NIR PeLEDs with device lifetimes meeting the demands of commercial applications [108]. However, the operational lifetimes of PeLEDs emitting in the visible range remain unsatisfactory [4, 15, 94]. Ion migration and phase instability are some of the key reasons for device instability. High-performance blue [103, 109]- and white-emitting [110, 111] devices are yet to be demonstrated, which are essential for display and solid-state lighting applications. Moreover, issues concerning efficiency roll-off [112] and lead toxicity in high-performance PeLEDs require further attention from researchers in the field.

### Current Progress of Perovskite LEDs

#### Material Design

The optoelectronic properties of perovskite emitters can be modulated by dimensional and compositional engineering [126]. Through dimensional engineering, perovskite emitters with various forms of 3D bulk crystals, 2D/quasi-2D layered crystals or nanosheets, 1D nanowires and 0D quantum dots can be successfully fabricated by controlling the synthetic chemistry [127]. Modulating the dimensionality of perovskite is an effective method to control the emissive color and obtain desired optoelectronic properties like high radiative efficiency, excitonic/non-excitonic nature, charge/energy transfer routes and anisotropic emission [126]. Through mixing the X-site halide anions of I^−^, Br^−^ and Cl^−^, emission from violet (ABCl_3_), to blue (ABCl_x_Br_3 − x_), to green (ABBr3), to red (ABBr_x_I_3 − x_) and to near infrared (ABI_3_) can be achieved, facilitating a wide range of applications including display and lighting [111, 128]. In addition, modulating A-site and B-site cations also contributes to the improvement of crystal quality and reduction of perovskite toxicity.

#### Charge Carrier Dynamics

As the excited-state carriers in 3D and low-dimensional (2D/1D/0D) perovskites tend to show non-excitonic and excitonic characters (Fig. 3b), respectively, free carriers and excitons are likely to form in these two systems accordingly. Therefore, their charge carrier dynamics can be different and are discussed separately as below [129]:

1 $$-\frac{{\text{d}}{\text{n}}_{\text{fc}}}{{\text{d}}{\text{t}}}\;{=}\;{\text{k}}_{1}{{\text{n}}}_{\text{fc}}\;{+}\;{\text{k}}_{2}{{\text{n}}_{\text{fc}}}^{2}\;{+}\;{\text{k}}_{3}{{\text{n}}_{\text{fc}}}^{3} \, \text{ (Non-excitonic system)}$$

2 $$-\frac{{\text{d}}{\text{n}}_{\text{ex}}}{{\text{d}}{\text{t}}}\;{=}\;{\text{k}}_{1}{{\text{n}}}_{\text{ex}}\;{+}\;{\text{k}}_{2}{{\text{n}}_{\text{ex}}}^{2} \, \text{ (Excitonic system)}$$

where *n*_fc_ and *n*_ex_ are free carrier and exciton density, respectively; *k*_*1*_ is the decay rate constant of monomolecular recombination [for low-dimensional perovskites, *k*_*1*_ is the total decay rate of trap-assisted recombination (*k*_trap_) and germinated recombination of the exciton (*k*_ex_), i.e., *k*_*1*_ = *k*_trap_ + *k*_ex_; for 3D perovskites, *k*_*1*_ is dominated by the decay rate of trap-assisted recombination due to the absence of exciton, i.e., *k*_*1*_ ≈ *k*_trap_];* k*_*2*_ and *k*_*3*_ are the decay rate constants of bimolecular recombination and Auger recombination, respectively.

In 3D perovskites, trap-assisted recombination and Auger recombination are typically non-radiative processes, while bimolecular recombination is intrinsically radiative. In low-dimensional perovskites, trap-assisted recombination and bimolecular recombination (also known as exciton–exciton annihilation or Auger recombination) are non-radiative processes, while germinated recombination of the exciton is radiative. In such a case, the photoluminescence quantum yields (PLQY) of these two kinds of perovskites can be described as:

3 $${\text{PLQY}} = \frac{{k_{2} n_{{{\text{fc}}}} }}{{k_{1} + k_{2} n_{{{\text{fc}}}} + k_{3} n_{{{\text{fc}}}} ^{2} }}({\text{Non}} - {\text{excitonic}}\;{\text{system}})$$

4 $${\text{PLQY}} = \frac{{k_{{{\text{ex}}}} }}{{k_{{{\text{ex}}}} + k_{{{\text{trap}}}} + k_{2} n_{{{\text{ex}}}} }}({\text{Excitonic}}\;{\text{system}})$$

In terms of quasi-2D perovskites which generally consist of excitonic and non-excitonic perovskite components, charge carrier dynamics become complicated and the field tends to simplify the discussion by considering this system as an excitonic system (if the system is predominantly excitonic) or non-excitonic system (if energy/charge transfer from low-dimensional perovskites to high-dimensional perovskites are efficient).

Note that the aforementioned discussion is typically based on the photoexcitation process. However, there are some discrepancies between photoexcitation and charge injection. Having a high PLQY does not necessarily mean that a PeLED will achieve a high EQE, so it is also critical to understand the carrier dynamics of the device. For PeLEDs, the high electron injection efficiency and low hole injection efficiency lead to charge injection imbalance, which severely affects the performance of PeLEDs. At low current densities, more radiative recombination can be maintained with less carrier injection. At high current densities, the number of carriers injected increases and exciton bursts are prone to occur between the HTL and the emitter layer (EML) due to energy level mismatch. Lin et al. established the “energy ladder,” which effectively improved the hole injection and mitigated the carrier injection imbalance problem [130]. Wei et al. introduced an insulating layer of poly(methyl methacrylate) (PMMA) between EML and ETL, which blocked the excess injection of electrons and facilitated the balance of electron and hole injection. The efficiency of the device was greatly improved, with EQE over 20% and stability over 100 h (*T*_50_ > 100 h) [94]. Zeng et al. obtained efficient white light by regulating the excited-state process through a photoelectric synergistic strategy [110]. Understanding excited-state processes and effective modulation strategies are expected to facilitate further optimization of high-performance LEDs.

#### Device Architectures

Typically, PeLEDs device architecture comprises intrinsic emitting layer in a double-heterojunction structure with P-type HTLs and N-type ETLs. Upon forward bias, holes are injected into the top of the valence band (VBM) of the EML via the highest occupied molecular orbital (HOMO) of the HTL; while electrons are injected into the conduction band bottom (CBM) of the EML via the lowest unoccupied molecular orbital (LUMO) of the ETL. Subsequently, light emission originates from exciton recombination or bimolecular recombination in the EML. The energy difference between VBM and CBM of EML determines the wavelength of the emitted photons. Well-designed device architecture plays a key role in high-efficiency PeLEDs [131]. Balanced charge carrier transport is one of key prerequisites to maximize the IQE of a PeLED. Mismatched injection will cause carrier accumulation at the interface and excess current without radiative contribution, reducing the IQE and EQE (Fig. 3c) [132].

Adjusting energy level alignment is an important part in device architecture designs. Perovskite layers exhibit tunable bandgap, with VBM approximately varying from − 5.4 to − 6.11 eV and the CBM around − 3.30 eV for near-infrared emission [123, 132]. The commonly used ETL, 2,2′,2″-(1,3,5-benzinetriyl)-tris(1-phenyl-1-H-benzimidalzole) (TPBi), has a LUMO level of − 2.8 eV [131]. The typical HTL poly(3,4-ethylenedioxythiophene):polystyrene sulfonate (PEDOT:PSS) has a HOMO level of ~ 5.2 eV, which will cause less efficient hole injection [133]. Reducing hole injection barrier in PeLEDs with various colors, especially for blue PeLEDs, is critical to achieve high quantum efficiency.

Modulating the work function (WF) and designing graded energy levels are widely employed approaches to improve charge injection into HTLs. Introducing molecules with high ionization potential into HTL is able to induce a shift of vacuum level and modulate the WF of HTL [134]. It is reported that adding a perfluorinated polymeric acid into PEDOT:PSS can induce a self-organized buffer hole injection layer, which exhibits gradually increased WF (from 5.2 to 5.9 eV) [135]. Through offsetting the hole injection barrier, the EQEs of green and red PeLEDs reached over 20% [123, 136]. However, the HOMOs of conventional monolayer HTLs do not match the VBMs of pure red and blue perovskites. Establishing graded HOMO energy levels using multilayered HTLs is another solution. To achieve this, organic buffer layers have been deposited on top of the PEDOT:PSS layer, such as poly[N,N’-bis(4-butylphenyl)- N,N’-bisphenylbenzidine] (poly-TPD), poly(9-vinycarbazole) (PVK) and poly(9,9-dioctylfluorene-alt-N-(4-s-butylphenyl)-diphenylamine) (TFB) [123]. Sargent et al. employed PEDOT:PSS/poly-TPD multilayered HTLs to obtain pure red PeLEDs with EQE up to 24.4% and emission located at ~ 650 nm [137]. Yuan et al. utilized Pedot:PSS/PVK/PVP as multilayered HTLs and achieved sky-blue PeLEDs with EQE of 14.2% (emission located at 475 nm) [138]. However, when constructing multilayered HTLs, the interfacial characteristics, including contact barriers, charge scattering, defects and wettability, should be considered [139].

#### Interfacial Control

Emissive layer/charge transport layer interfaces play a key role in the performance of PeLEDs, as interfacial traps are a dominant channel for non-radiative recombination losses [140]. Buried interfaces can act as templates for the nucleation and crystallization of perovskites, modulating the crystallinity, morphology, trap density and work functions of the perovskites [101, 104]. It was reported that ultrathin polar interfaces including lithium fluoride (LiF) could enable the formation of highly emissive and uniform perovskite films on hydrophobic polymeric charge transporters in an OLEDs-like device configuration [104]. This led to the demonstration of efficient green quasi-2D PeLEDs with EQEs of up to 19.1% at > 1500 cd m^−2^. Similarly, hydrophilic interfaces prepared using polymers such as polyethyleneimine (PEI) [101], perfluorinated ionomer (PFI) [141] and small molecules including ethanolamine (ETA) [142], aluminum oxide (Al_2_O_3_) [143], were found to allow the formation of high-quality perovskite films. Although these strategies have been proved useful for high-performance PeLEDs, further mechanistic investigations are required to reveal the origins of such improvements.

Besides the modification of the buried interfaces, engineering top interfaces might have additional advantages in surface defect passivation and inhibition of ion migration [117, 118]. An early example of top surface modification was the post-treatment of perovskite emissive layer using trimethylaluminum (TMA) vapor in an atomic layer deposition (ALD) tool. The resultant interfacial passivating layer enhanced solvent resistance of the perovskite, improving device EQEs from 0.2% to 5.7% [117]. Compared to bottom surface engineering, top interface modifications present additional challenges including surface reconstruction, residual solvents and orthogonal solvent deposition of functional layers on top of the interfaces.

Bifacial passivation strategies capable of treating both bottom and top interfaces of the perovskite emissive layers were explored, enabling a peak EQE of 18.7% and improved stability for quantum dot PeLEDs [96]. Apart from the aforementioned effects, interfacial control can provide additional benefits in optimizing charge balance for efficient PeLEDs [94, 104].

#### Light Outcoupling

Non-radiative recombination processes within the PeLEDs emissive layers have been effectively suppressed, leading to IQEs approaching 100% [93]. The EQE of a PeLED is related to its IQE through EQE = *η*_out_ IQE, where *η*_out_ is the light outcoupling efficiency. *η*_out_ is presumed to be ~ 20% for an OLEDs-like device structure. Opportunities in improving EQE beyond this value lie in the ability of extracting trapped photons from the PeLEDs. Light outcoupling strategies for PeLEDs can be briefly divided into two categories, modifications of the perovskite emissive layer properties and the employment of external optical structures.

Notable light outcoupling approaches based on tailoring of the perovskite emissive layer properties include the reduction of refractive index (*n*) [93, 144], control of transition dipole moment orientations [114, 145], formation of light scattering structures within the emissive layer [92] and photon recycling [107, 146]. According to the ray optics limit of 1/2*n*^2^, the reduction of *n* can increase *η*_out_ for planar PeLEDs. For typical 3D perovskites, *n* is ~ 2.5. Including 2D ligands with a significant fraction of organic content in the perovskite composition lowers *n* to ~ 2.1 [144]. Introducing polymers into the perovskite emissive layers was reported to reduce *n* further to ~ 1.9 [93], resulting in an *η*_out_ of ~ 21% and EQEs of up to 20.1% [93]. Orientation of transition dipole moments (TDMs) also plays a critical role in the light outcoupling processes, as only emission from horizontally oriented TDMs can be effectively extracted. The fraction of horizontal TDMs could be tuned by engineering the perovskite nanostructures [114, 147]. For PeLEDs based on nanoplatelets [114], a horizontal dipole fraction of ~ 84% and *η*_out_ of 31% were reported. Tuning of the fraction of horizontal TDMs can also be achieved for solution-processed polycrystalline perovskite films [147]. Light scattering in rough or structured emissive layers was expected to enhance *η*_out_. PeLEDs based on submicrometer-scale structures were reported to have outcoupling efficiencies of ~ 30%, leading to peak EQEs of up to 20.7% [92]. Photon recycling improves the light extraction from PeLEDs by randomizing the directions of trapped photons in a fashion similar to scattering, but through the reabsorption and re-emission of light [146]. It was reported that for PeLEDs based on perovskite emitters with high internal radiative efficiencies and small Stokes shifts (e.g., PEA_2_Cs_n − 1_Pb_n_Br_3n + 1_), about 30–70% of EL may originate from photon recycling [107].

External optical structures such as lenses [15, 148] and microcavities [149] are capable of converting optical power in substrate and waveguide modes into outcoupled modes, resulting in substantially increased *η*_out_. It was reported that a light outcoupling hemispherical lens improved the peak EQEs of perovskite nanocrystal LEDs from 23.4% to 45.5% [15]. Microcavities were used to improve *η*_out_ from ~ 20% to ~ 30% for top-emitting PeLEDs featuring transparent electrodes, leading to peak EQEs of up to 20.2% [149]. Similarly, other light outcoupling approaches employing plasmonic effects [150, 151] and refractive index matching [152] were found to improve *η*_out_ for PeLEDs.

### Challenges Ahead

#### Device Stability

Similar to perovskite solar cells, the poor operational stability of PeLEDs stands as the biggest challenge toward commercial applications. Halide perovskites were considered intrinsically unstable under electric fields due to the soft and ionic nature of their crystal lattices (Fig. 3d) [153–157]. Typical device lifetimes (*T*_50_) of PeLEDs range from 1 to 100 h, far from that required for commercial applications (> 10^4^ h at practical photon fluxes) [5, 92, 94, 97, 158]. A recent breakthrough in this area was the demonstration of NIR PeLEDs with EQEs of up to 22.8% and ultralong life spans, enabled by a dipolar molecular stabilizer which inhibited ion migration at grain boundaries [108]. The PeLEDs exhibited no clear degradation over 5 months of continuous operation at 5 mA cm^−2^. From accelerated aging tests, T_50_ lifetimes were estimated to be ~ 1.2 × 10^4^ h and ~ 3.3 × 10^4^ h at 5 mA cm^−2^ (~ 3.7 W sr^−1^ m^−2^) and 3.2 mA cm^−2^ (~ 2.1 W sr^−1^ m^−2^), respectively (Fig. 3e, Table 4). Longer *T*_50_ lifetimes of up to 2.4 × 10^6^ h were estimated for lower current densities. These results could alleviate the critical concern that halide perovskite devices may be intrinsically unstable, paving the path toward industrial applications. Despite these encouraging results, stable PeLEDs emitting in the visible spectral range is yet to be demonstrated [115, 119–121, 123].

**Table 4:** Lifetime of the state-of-the-art PeLEDs

Emissive layer	Device structure	Operational condition	T_50_ lifetime (h)	References
FAPbI_3_: SFB10	ITO/ZnO/PEIE/Pero/TFB/MoO_x_/Au	0.7 mA cm^−2^, ~ 0.21 W sr^−1^ m^−2^	2.4 × 10^6^ (extrapolated)	[108]
		3.2 mA cm^−2^, ~ 2.1 W sr^−1^ m^−2^	32,675 (extrapolated)	
		5 mA cm^−2^, ~ 3.7 W sr^−1^ m^−2^	11,539 (extrapolated)	
		10 mA cm^−2^, ~ 8.1 W sr^−1^ m^−2^	2984	
		20 mA cm^−2^, ~ 16.5 W sr^−1^ m^−2^	877.1	
		100 mA cm^−2^, ~ 69.6 W sr^−1^ m^−2^	120.3	
		200 mA cm^−2^, ~ 120 W sr^−1^ m^−2^	22.4	
FAPbI_3_: PAC	ITO/ZnO/PEIE/Pero/TFB/MoO_x_/Au	20 mA cm^−2^, ~ 17 W sr^−1^ m^−2^	682	[124]
CsPbI_3_: PMA	ITO/PEDOT:PSS/poly-TPD/Pero/TPBi/LiF/Al	30 mA cm^−2^, ~ 150 cd m^−2^	300	[119]
CsPbI_3_ QDs: perovskite matrix	ITO/ poly-TPD/LiF/Pero/TPBi/LiF/Al	20 mA cm^−2^, 914 cd m^−2^	25	[123]
		~ 2 mA cm^−2^, ~ 100 cd m^−2^	2100 (extrapolated)	
CsPbBr_3_: PEABr:MBA:K_2_S_2_O_8_	ITO/PVK/F4TCNQ/Pero/TPBi/LiF/Al	0.2 mA cm^−2^, 100 cd m^−2^	208	[120]
CsPbBr_3_: CsTFA	ITO/PEDOT:PSS/Pero/TPBi/LiF/Al	~ 1 mA cm^−2^, 100 cd m^−2^	250	[125]
CsPbBr_3_ QDs	ITO/PEDOT:PSS/PVK/Pero/ZnCl_2_/Pero/Ag	7.5 mA cm^−2^, 100 cd m^−2^	59.2	[121]
CsPb(Br/Cl)_3_: PEACl:YCl_3_	ITO/PEDOT:PSS/Pero/TPBi/LiF/Al	3.2 V, 100 cd m^−2^, ~ 1 mA cm^−2^	~ 1.6	[115]

Ion migration is a primary factor limiting the lifetimes of PeLEDs [155]. Under external stimuli including electric fields, heat and light, ion migration can occur due to the low ion migration activation energy and the hybrid electronic–ionic conduction characteristics of the perovskites (Fig. 3d) [153–157]. The mobile ions can induce detrimental effects on PeLEDs through defect generation, lattice deformation, ion accumulation, ionic doping and chemical interactions [154, 156]. To suppress ion migration, strategies including molecular passivation [92, 94, 119, 120, 124, 125, 159, 160], dimensionality modulation [97, 115, 136, 161], thermal management [89] have been explored. It was reported that a cross-linking strategy using methylene-bis-acrylamide could effectively increase Br- binding energy and activation energy in the perovskite, leading to a *T*_50_ lifetime of 208 h [120]. As halide ions are generally considered to be the main contributor to ionic movements [120, 154, 157, 160, 162], further efforts in the management of halide ions are expected to show clear benefits. This may be achieved, for examples, by raising the barriers to halide migration with the aid of molecular stabilizers, reducing halide vacancies and developing perovskite materials with reduced ionic conductivities.

Phase transformation and halide segregation are key mechanisms for the instability of blue and red PeLEDs based on mixed-halide [162], mixed-dimensional perovskites [161] and perovskite emitters with undesirable tolerant factors [159, 163]. These processes may be triggered by unfavorable dimensionality distribution [161], halide migration [162], lattice strain/stress and externally induced structural evolutions [159, 163]. Halide segregation could be partially suppressed by treating nanocrystals with multidentate ligands, inhibiting the formation of iodine Frenkel defects [160]. Despite such improvements, the short *T*_50_ lifetime of 30 min at an initial luminance of 141 cd m^−2^ for these devices indicates that challenges in operational stability are still present for mixed-halide PeLEDs [160]. Issues including interfacial chemical interactions [124, 163] and thermal degradation [124] are additional contributors to structural instability. In situ structural characterizations during device operation may help to gain further insights into the degradation processes.

For reference, for high-efficiency OLEDs based on Ir(ppy)_3_, a luminance of 1000 cd m^−2^ corresponds to a radiance of 2.1 W sr^−1^ m^−2^, and a luminance of 100 cd m^−2^ corresponds to a radiance of 0.21 W sr^−1^ m^−2^.

#### Blue PeLEDs

With the realization of high-efficiency green, red and near-infrared PeLEDs, the inferior performance of blue PeLEDs has become increasingly prominent, especially for deep and pure blue with emission wavelengths shorter than 470 nm. A combination of the lower emission efficiency of the wide-bandgap emitters and the mismatched energy levels in the devices result in the undesirable performance of blue PeLEDs [164]. Figure 3f summarizes the feasible strategies to improve the performance of blue PeLEDs.

Adjusting halide anion species is a straightforward strategy to realize tunable emission. For instance, the blue-emitting perovskites can be obtained by mixing bromide and chloride precursors. Nevertheless, the increase of the chloride component will lead to a significant reduction of PLQY due to the lower formation energy of chloride vacancy defects [165]. Furthermore, the introduction of the chloride component will induce lattice distortion and reduce the Goldschmidt tolerance factor of the perovskite, resulting in unstable lattice structures [166]. The vacancy defects and unstable lattice structures will aggravate ion migration in the mixed bromide–chloride perovskite and deteriorate the spectral stability. To date, ion doping and defect passivation are typical strategies for suppressing ions migration and stabilizing emission spectra [109, 115, 133].

Another strategy for realizing blue emission is through low-dimensional pure bromide perovskite: quasi-2D perovskites and strongly confined nanocrystals [103, 113]. Low-dimensional perovskite has the potential to gain high PLQY due to its large exciton binding energy. The emission wavelength of quasi-2D perovskite mainly depend on the perovskite phase with the lowest bandgap, due to fast charge or energy transfer [112]. Therefore, it is necessary to control the phase distribution of perovskite. To enable stable blue emission, predominant *n* = 2 and *n* = 3 phases need to be achieved [113]. Rational composition design through adding large organic cations can achieve crystallization control of the quasi-2D perovskite and obtain uniform phase distribution [136]. For strongly confined nanocrystals, surface modification and ligand exchange are required to realize stable blue emissions [121, 167, 168]. Notably, nanocrystal size strongly influence the emission of nanocrystals. Large nanocrystal size distrubution will lead to wide emission, lowering the color purity. Further exploration of the nucleation and growth mechanisms of perovskite nanocrystals is required to achieve blue PeLEDs with high color purity.

So far, the reported blue PeLEDs with EQE > 10% all emit in sky-blue region (475—495 nm), which fails to meet the requirements of high-definition display [116, 122]. Developing efficient pure and deep blue PeLEDs (emission below 470 nm, meeting Rec. 2020 standards) is still a big challenge [169].

#### White PeLEDs

Conventional R/G/B mixed or stacked structure white PeLEDs (W-PeLEDs) show poor efficiency, which can be attributed to the inferior optoelectronic properties, solvent incompatibility and ion migration under electric field. So far, W-PeLEDs are usually achieved by means of self-trapping excitons (STEs) (Fig. 3g), ionic doping and organic/inorganic hybrid. The electrons and holes are easily captured due to lattice deformation and soft lattice characteristics, which eventually lead to the energy transfer of free excitons to STEs and the realization of broader spectra. Such phenomenon was observed in double perovskite and copper-based perovskite [170, 171]. Although poor carrier transport characteristics restrict device efficiency, its color rendering index (CRI) above 90 is superior to most materials. The use of organic additives is an effective strategy to enhance carrier injection and transport. Chen et al. improved the EQE to 3.1% by polymer doping [172]. The phase composition of CsPbI_3_ is temperature dependent. Zeng et al. reported uniformly distributed *α*- and *δ*-CsPbI_3_ by adjusting annealing time [110]. The combination of the broad-emission STEs in *δ*-CsPbI_3_ with the strong carrier transport in *α*-CsPbI_3_ creates an equilibrium state through energy transfer between the two phases. As a result, the carrier transport in *δ*-CsPbI_3_ is effectively enhanced, and the W-PeLEDs achieved a maximum EQE of 6.5% and a highest luminance of 12,200 cd m^−2^.

Heterogeneous ionic doping, such as Mn^2+^ and rare elements, can broaden the emission spectrum of perovskite materials and achieve white emission. The energy level position of the doped ions is the crux to the final spectral coverage [173–175]. Sun et al. combined the ionic spectra of lanthanide elements Sm^3+^ with that of blue CsPbCl_3_ NCs to achieve full visible spectral emission under different driving voltages [175]. Due to efficient and stable energy transfer from CsPbCl_3_ to Sm^3+^, the device exhibited superior EL spectral stability and a CRI as high as 93. Furthermore, perovskite/organic hybrid WLEDs are great candidates for highly efficient WLEDs. Liu et al. fabricated perovskite/organic hybrid WLEDs by using pure red-emissive perovskite and sky-blue-emissive organic p–i–n heterojunction as emissive layers. The heterojunction was achieved by embedding an ultrathin phosphorescent interlayer between p-HTL and n-ETL. The inserted p-HTL can perfectly suppress energy transfer between the perovskite and the phosphorescent interlayer and regulate the exciton recombination region, thus obtaining W-PeLEDs with EQEs up to 7.35% [176].

#### Efficiency Roll-off

Efficiency roll-off refers to the phenomenon that EQE decreases with the increase of current density, which becomes a general issue occurring in PeLEDs, retarding the progresses of efficient high-brightness devices and lasing.

As displayed in Fig. 3h, one main process accounting for efficiency roll-off is the non-radiative Auger recombination that dominates the total recombination at the high-carrier-density region, as its decay rate is in quadratic (*k*_*3*_*n*_fc_^*2*^) or linear (*k*_*2*_*n*_ex_) relationship with the carrier density in 3D or low-dimensional perovskites, respectively. In general, low-dimensional perovskites or 3D perovskites with smaller crystal sizes suffer from severer Auger recombination due to the confinement of carries within the limited physical space, resulting in a high local carrier density [177, 178]. Therefore, a straightforward way to suppress the Auger recombination is to enlarge the physical volume where recombination happens [112]. In addition, according to Fermi’s golden rule, the possibility of inter-/intra-band transitions between states positively correlate with their wave function overlapping. Therefore, reducing such a wave function overlapping (e.g., via constructing a core–shell structure for perovskite nanocrystals) also facilitates the reduction of the Auger decay rate [179].

Besides the intrinsic Auger loss, other factors exacerbating the device working conditions at the high-carrier-density region also led to efficiency roll-off. For example, the considerable Joule heat generated in devices might degrade the perovskite emitters [180]; the ion migration under high applied voltages might result in phase segregation and defect formation [181]; the leakage of carriers caused by the imbalance of injected electrons and holes [130].

#### Low-toxicity Emitters

The toxicity of Pb in lead halide perovskites has raised environmental concerns that could hinder the practical applications of PeLEDs [182]. Recently, great efforts have been devoted to seeking less toxic perovskite alternatives. Several strategies (Fig. 3i) have been demonstrated, including alloyed metal/Pb perovskites (such as Sn/Pb and Ge/Pb), tin halide perovskites, halide double perovskites and lead-free perovskite variants. The key of these strategies is partially or completely replacing Pb with other less or non-toxic elements (such as Sn, Ge, In, Bi, Sb and Cu) while ideally maintaining the perovskite structure and high performance.

Partial replacement of Pb by Sn or Ge is a useful approach for achieving reduced-toxicity perovskites without severely sacrificing performance. It has been demonstrated that CsPb_0.67_Sn_0.33_Br_3_ and PEA_2_Cs_n − 1_(Ge_0.1_Pb_0.9_)_n_Br_3n + 1_ perovskite films separately exhibited high PLQYs of 45% and 71%, resulting in efficient PeLEDs with EQE of 4.13% and 13.1%, respectively [183, 184]. However, a remaining challenge for these alloyed metal/Pb perovskites is the rapid decrease in PLQY with an increasing metal/Pb ratio.

Tin halide perovskites are a main class of lead-free perovskite materials because Sn possesses the most similar characteristics with Pb, such as same valence and similar ionic radii. Like their lead halide counterparts, tin halide perovskites have all-inorganic (e.g., CsSnI_3_) and hybrid organic–inorganic structures (e.g., CH_3_NH_3_SnI_3_ and (PEA)_2_SnI_x_Br_4 − x_) [185, 186]. Although these materials typically experience the problems of Sn oxidation and rapid crystallization which cause severe non-radiative recombination [187], tin halide PeLEDs have demonstrated decent EQEs up to 5.4% [188]. It is also worth noting that two-dimensional (2D) tin halide perovskites show superior properties, including efficient charge injection, strong emission at room temperature and high stability, which are favorable for fabricating significantly better PeLEDs than lead halide counterparts [189].

Halide double perovskites, with a formula of A_2_B^I^B^III^X_6_, represent another rich material library for lead-free perovskites. Although many material choices can be anticipated with different B^I^ and B^III^ combinations, most of them show low PLQY because of the indirect-bandgap nature or parity-forbidden transition in direct-bandgap systems [190]. Metal ion doping has been demonstrated as a promising approach to tackle the low PLQY problem owing to the formation of STEs states [170, 190]. For instance, by introducing Bi^3+^ and Na^+^, Cs_2_Ag_0.6_Na_0.4_InCl_6_:0.04% Bi powders showed a PLQY of ~ 86%, three orders of magnitude higher than that of Cs_2_AgInCl_6_ [170]. However, fabricating efficient PeLEDs based on halide double perovskites remains very challenging, because of the low quality of the perovskite films [170].

Lead-free perovskite variants, including A_3_B_2_X_9_-type materials [191] and non-perovskite metal halide compounds (such as Cs_3_Cu_2_I_5_ and CsCu_2_I_3_) [192, 193], are also promising alternatives to lead halide perovskites. Notably, copper halide films showed high PLQY over 80% and delivered a record-high EQE of 7.4% in LEDs [192].

To conclude, the current limitations in the development and commercialization of PeLEDs are the poor blue and white PeLEDs efficiency, unstable spectra, operating lifetime and toxicity of lead ions. While device efficiency, stability and the preparation of low-lead perovskite can be improved by materials design, ion doping, charge carrier dynamics regulation, device architecture engineering, interfacial control and light outcoupling techniques. More efforts are still needed in this area.

## Perovskites for Photodetection and Imaging

### Fundamentals

Photodetectors (PDs) are the essential component of various modern photodetection and imaging techniques, including spectroscopy, optical fiber communication, silicon-based complementary metal oxide semiconductor (CMOS) image sensing, light detection and ranging, X‑ray imaging, biomedical imaging, etc. Photodetection processes generally involve photon absorption, carrier generation and extraction, signal storage, data processing and subsequent signal reconstruction, which convert incident optical signals that could carry the modulated photon information into processable electrical signals.

### Current Progress

#### Visible-blind UV Detection

Wide bandgap chlorine-based perovskites have been demonstrated as promising candidates in visible-blind UV photodetectors and imaging arrays [8, 194–196]. Bakr et al., for the first time, grew high-quality MAPbCl_3_ single crystals with the dimension of 2 mm × 4 mm × 4 mm via inverse temperature crystallization and applied them in visible-blind UV detection [194]. The grown single crystals exhibited a bandgap of 2.88 eV, a trap density of ~ 3.1 × 10^10^ cm^−3^, a carrier mobility of ~ 42 ± 9 cm^2^ V^−1^ s^−1^ and a best-case diffusion length of ~ 8.5 μm, which are comparable with that of MAPbI_3_ and MAPbBr_3_ single crystals. The fabricated UV photodiode showed a detectivity of 1.2 × 10^10^ Jones and a responsivity of 46.9 mA W^−1^ under 365 nm light illumination. Since then, perovskite-based visible-blind UV photodetectors began to flourish by adopting perovskites with various morphologies in different device structures [194, 195, 197–200]. Particularly, Sargent et al. grew a dense layer of interconnected MAPbCl_3_ single crystals atop two adjacent ITO electrodes to fabricate ITO/MAPbCl_3_/ITO photoconductors, which achieve a responsivity of 18 A W^−1^ below 400 nm wavelength and an imaging-compatible response time of 1 ms, resulting from the short device length of 5 μm and the long carrier lifetime [195]. To further achieve UV–C (200–280 nm) detection and imaging, Zhou et al. creatively combined CsPbBr_3_ quantum dot fluorophor with a uniform MAPbI_3_ perovskite photodiode array to realize UV down-conversion photodetectors [196]. The layer of CsPbBr_3_ quantum dot fluorophor effectively converted UV-C light into visible light for the perovskite photodiode array to detect and simultaneously acted as a protective layer to avoid the fast UV-induced degradation of perovskites. As a recent breakthrough, Liu et al. firstly grew a CsPbCl_3_ polycrystalline layer via sequential vapor deposition technique and fabricated a large-area and uniform UV image sensor with 625 pixels, exhibiting great imaging capability [8].

#### Visible Detection

The bandgaps of perovskites can be tuned via halide composition engineering, making them suitable for visible photodetection. Back in 2014, Yang et al. innovatively demonstrated MAPbI_3 − x_Cl_x_ as the photoactive layer in a photodiode, whose spectral response range covered the whole visible region. Notably, the perovskite-based photodetectors performed better than most of the organic, quantum dot and hybrid counterparts, and show comparable merits with traditional inorganic semiconductor-based counterparts in the same period [10]. Since then, numerous strategies, including device structure design, surface passivation, bulk doping, band alignment and buffer layer engineering, have been developed in an attempt to improve the detection performance [201–204]. Bakr et al. grew large-area MAPbBr_3_ single-crystal films on top of two separated ITO electrodes via anti-solvent vapor-assisted crystallization method. The fabricated planar-integrated photoconductors show an ultrahigh responsivity (over 4 × 10^3^ A W^−1^ within 400–500 nm wavelength), a high gain (above 10^4^ electrons/photon) and a high gain–bandwidth product (above 10^8^ Hz) [205]. In addition to the broad spectral response, perovskite photodetectors can also achieve filter-free tunable narrowband photodetection [206, 207]. Huang et al. achieved narrowband photodetection with a full width at half maximum of less than 20 nm using perovskite single-crystal photodetectors and the response spectra of which could be continuously tuned from ~ 425 to ~ 650 nm by composition engineering [206]. Moreover, the nature of flexibility of perovskite photodetectors further extend their application scenarios to flexible sensing devices. Pan et al. fabricated CsPbBr_3_ film-based photodetector arrays (10 × 10 pixel) with precise pixel position, controllable morphology and homogenous dimension via vacuum-assisted drop-casting patterning techniques [208]. The waterproof parylene-C substrate also worked as the encapsulation layer to guarantee their long-term stability. This ultrathin photodetector arrays showed a potential application in artificial vision sensing.

#### Infrared Detection

Recently, the spectral response range of perovskite photodetectors has been extended to near- and mid-infrared wavelengths through sub-bandgap absorption, the combination with narrow-bandgap materials and tin–lead binary mixtures [209]. Despite the relatively large bandgaps, MAPbI_3_ perovskite bulk single crystals exhibit a certain degree of NIR detection capability through sub-bandgap absorption [210, 211]. Meredith et al. deposited interdigitated electrodes onto the surface of MAPbI_3_ single crystals to make photoresistors, which exhibited up to 900 nm wavelength NIR response owing to their surface trap-state-induced sub-bandgap absorption [210].

Incorporating narrow-bandgap materials is a common approach for Pb-based perovskites to achieve near- and mid-infrared response, including organic materials, silicon, germanium and two-dimensional materials [212–214]. Shi et al. pioneered to combine MAPbI_3_ film with a polymer film of PDPP3T to broaden the spectral response range of photodetectors. A responsivity of 5.5 mA W^−1^ and a specific detectivity of 3.2 × 10^9^ Jones at 937 nm wavelength under 1 V bias was achieved [212]. It is worth mentioning that this perovskite/polymer-based photodetectors exhibit an excellent flexibility and durability with the responsivity remaining 85% after 1000 bending cycles at a curvature radius of 7 mm. To further expand the response region to telecommunication wavelengths, a 300 nm germanium layer was incorporated to form a germanium/perovskite heterostructure. The fabricated device can achieve broadband detection from visible to infrared telecommunication band, with a responsivity of 1.4 A W^−1^ at 1550 nm wavelength, a specific detectivity of 10^8^ Jones and a rise/fall time of 2.1/5.7 ms [213]. Two-dimensional materials (graphene, PtSe_2_, PdSe_2_, MoS_2_, etc. [9, 214–216]) have also been widely utilized in perovskite-based NIR photodetectors in recent years due to their high carrier mobility and thickness-dependent bandgaps. Tsang et al. integrated Cs-doped FAPbI_3_ with two-dimensional PdSe_2_ to develop a fast, self-powered and broadband photodiode, with a wide response range of 200–1550 nm and a fast response speed of 3.5/4 µs for rise/fall time, respectively [9].

Partially substituting Pb^2+^ with Sn^2+^ to grow Sn–Pb mixed perovskites can narrow down the bandgap to ~ 1.17 eV, which directly convert NIR optical signals into electric signals [217–219]. Typically, Choy et al. [219] effectively controlled film crystallization kinetics and successfully grew Sn-rich binary perovskite films with intensified preferred orientation and decreased trap density. The enhanced quality of Sn-based perovskite films gave rise to a high responsivity of 200 mA W^−1^ at 940 nm for single photodetector device and excellent photocurrent uniformity for the 6 × 6 pixel arrays. Methods of reductant doping, component engineering, adopting suitable buffer layers and surface defects passivation also have been demonstrated to effectively enhance the performance and stability of Sn–Pb mixed perovskite-based infrared detection devices [217–221].

#### Gamma-ray Detectors

Gamma-ray (*γ*-ray) detectors have a wide range of applications, including medical imaging, homeland security, industrial inspection, nuclear industry, high-energy physics and astrophysical scientific research [222]. However, there are certain limitations for the existing *γ*-ray detectors, for example, inability to work at room temperature (high-purity germanium (HPGe)), poor radiation absorption (silicon (Si)) and high preparation costs (cadmium zinc telluride (CdZnTe)) [223–225]. In recent years, perovskites have been expected to be promising candidates for radiation detection materials by virtue of excellent optoelectronic performances [12, 226].

Perovskites have a series of beneficial properties that are desired for γ-ray detection. Firstly, the high ray attenuation coefficient of perovskites ensures the effective absorption of high-energy photons. Secondly, the large and balanced carrier mobility–lifetime (μτ) product for perovskite enables efficient charge collection [222]. Thirdly, the bandgap of perovskites (1.5–2.3 eV) is suitable. Typically, the small-bandgap materials have intrinsic low resistivity that would lead to large dark current and noise. While the resistivity of perovskites can reach 10^8^–10^10^ Ω cm through optimizing single-crystal processing and device design, enabling a low dark current [227–229]. Furthermore, an important advantage of perovskites is the large-area and low-cost processability. High-quality single crystals can be grown by both solution and melt processes [126, 230, 231]. The size of the crystals can achieve a few centimeters, and the cost (< $1.0 per cm^3^) is orders of magnitude less than that of CdZnTe [222, 226].

**Fig. 4:**
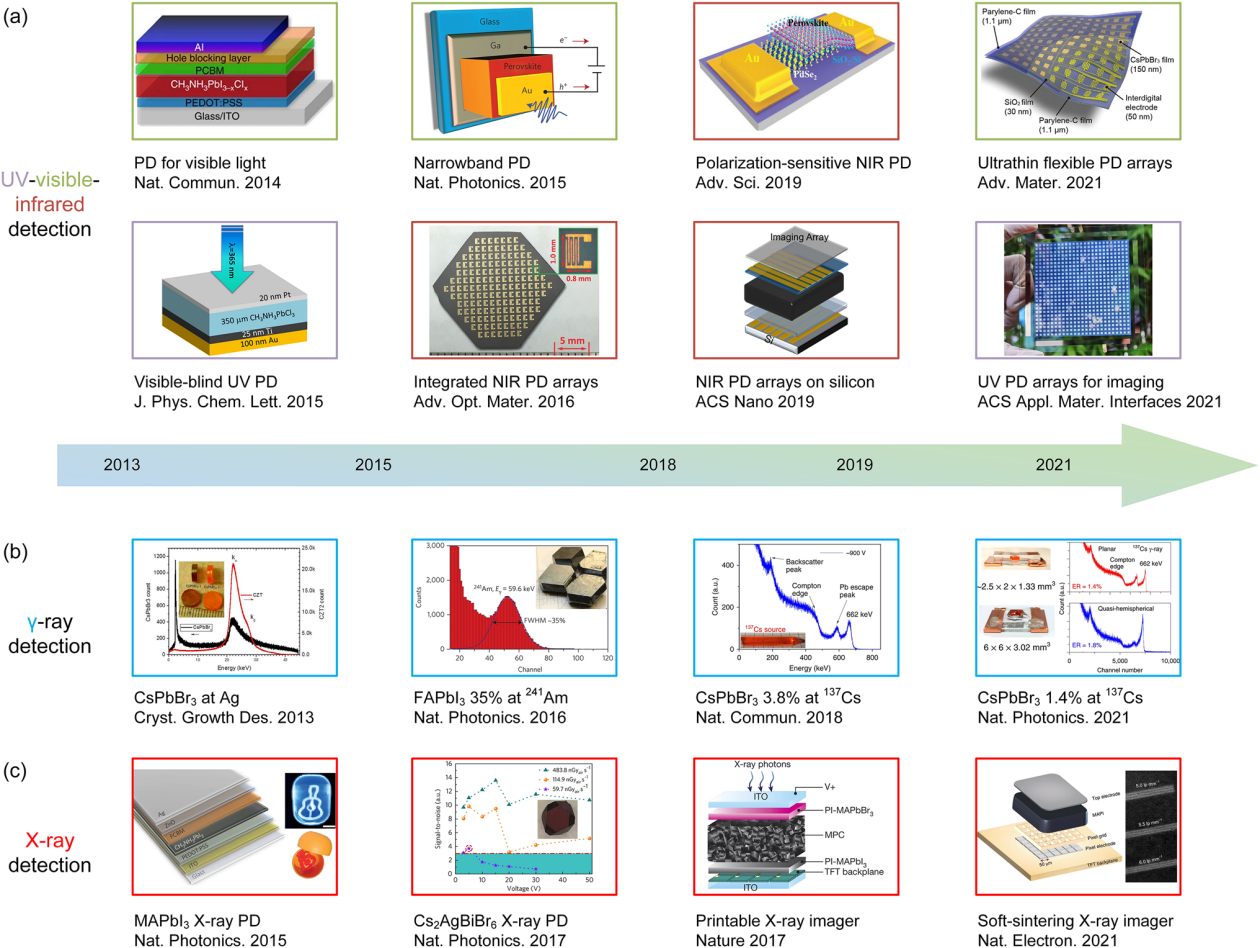
Timeline of perovskite **a** UV–visible–NIR photodetectors, Reproduced with permission [10], Copyright 2014, Springer Nature; [206], Copyright 2015, Springer Nature; [194], Copyright 2015, American Chemical Society; [211], Copyright 2016, John Wiley & Sons, Inc. [219], Copyright 2019, American Chemical Society; [9], Copyright 2019, John Wiley & Sons, Inc.; [208], Copyright 2021, John Wiley & Sons, Inc.; [8], Copyright 2021, American Chemical Society. **b** γ-ray detectors. Reproduced with permission [232], Copyright 2013, American Chemical Society; [233], Copyright 2016, Springer Nature; [229], Copyright, 2018, Springer Nature; [235], Copyright 2020, Springer Nature. **c** X-ray detectors. Reproduced with permission [236], Copyright, 2015, Springer Nature; [237], Copyright 2017, Springer Nature; [238], Copyright 2017, Springer Nature; [11], Copyright 2021, Springer Nature

Perovskites γ-ray detectors have been developed rapidly in recent years, as shown in Fig. 4. In 2013, Stoumpos et al. [232] first demonstrated the feasibility of perovskite radiation detector. The melt-grown CsPbBr_3_ single crystal resolved the X-ray peaks of Ag source (21.59 keV). In 2016, Yakunin et al. [233] presented the first example of γ-photon spectroscopic detection based on solution-grown FAPbI_3_ single crystals. The corresponding energy resolution of ^241^Am source (59.5 keV) was acquired as 35%. Moreover, the perovskite γ-photon counting detection was also investigated in this work, which showed potential to be applied in nuclear medicine. To enhance γ-ray energy resolution, many studies have been devoted to minimizing device noise and increasing charge collection efficiency. Through component engineering, Wei et al. [228] grew MAPbBr_2.94_Cl_0.06_ single crystal with high resistivity of 3.6 × 10^9^ Ω cm and large μτ product of 1.8 × 10^−2^ cm^2^ V^−1^. The fabricated detectors realized 6.5% energy resolution for 662 keV ^137^Cs *γ*-ray. Subsequently, He et al. [234] designed Schottky-type detectors with a structure of Ga/MAPbI_3_ single crystal/Au in 2018. Ga and Au electrodes can block the hole and electron injection from the anode and cathode, respectively, reducing the dark current. A better energy resolution (12%) of ^241^Am 59.5 keV was achieved by the Schottky-type MAPbI_3_ device. And for the higher-energy *γ*-photon ^57^Co 122 keV, the energy resolution was acquired as 6.8%. This Schottky-type structure had also made impressive progress on melt-grown CsPbBr_3_ single-crystal device. The device of Ga/CsPbBr_3_/Au successfully detected ^57^Co 122 keV and ^137^Cs 662 keV *γ*-rays, demonstrating an energy resolution of 3.9% and 3.8%, respectively [229]. In 2021, He et al. [235] further enlarged the scale of CsPbBr_3_-based detectors to 1.5 inches in diameter and adopted a unipolar hole-only sensing techniques (pixelated and quasi-hemispherical) to enhance the hole signal spatially. The small planar (6.65 mm^3^), quasi-hemispherical (109 mm^3^) and 2 × 2 pixelated (297 mm^3^) detectors achieved excellent energy resolution of 1.4%, 1.8% and 1.6%, respectively, at ^137^Cs 662 keV. Besides, CsPbBr_3_ detectors presented good thermal stability (from − 2 to 70 °C) and outstanding operation stability (over 18 months after encapsulation), shedding light on their practical applications.

There are still many technical and scientific issues remaining unsolved for perovskite *γ*-ray detectors. For photon counting detection and imaging, the detector needs to be well characterized at high photon flux, i.e., the photon count rate should be high [239]. When the photon flux exceeds the device count rate, the detectors will experience pulse pile-up, resulting in energy spectrum distortion [240]. Since the mobility of perovskite is relatively low (< 100 cm^2^ V^−1^ s^−1^), the photoresponse speed of the perovskites device is slow and the response time is as long as tens of microseconds [12, 241]. The count rate of CsPbBr_3_ detectors have been reported as 10^5^ to 10^6^ photons s^−1^ pixel^−1^, which is lower than the clinical medical ray flux of 10^9^ photons s^−1^ mm^−2^ [239, 241]. Thus, the mobility of perovskites needs to be increased to improve charge collection efficiency for photon counting application. Furthermore, the growth of high-quality perovskite single crystals with large size is still a major challenge. The defects, impurities and grain boundaries all can cause charge trapping in perovskite crystals, which may reduce the resolution and damage the energy response linearity [242]. The operation stability is another issue that needs to be emphasized. It is necessary to clarify and eliminate the perovskites detector polarization effect under high-dose radiation and high electric field [241]. In summary, the exciting achievement of perovskite *γ*-ray detection encourages further attempts to solve the above challenges and promote the innovation of semiconductor radiation detector at room temperature.

#### X-ray Detection and Imaging

X-ray has been widely used in nondestructive detections, enabling many applications in industrial inspection, security checks and medical examination [243–245]. MHP become very promising candidates for direct X-ray detecting materials owing to the large μτ product, strong X-ray absorbance and cost-effective processing [246, 247]. The very first perovskite-based direct X-ray detector adopted a solution-processed polycrystalline solar cell structure [236]. Then, many single-crystal-based perovskite X-ray detectors with high performance were reported, like CsPbBr_3_ and MAPbI_3_ [248, 249]. The lead-based 3D perovskites often exhibit superior charge collection efficiency but a relatively large dark current and strong ion drift. Later many 2D perovskites and 0D double perovskites including CsAgBiBr_6_, (NH_4_)_3_Bi_2_I_9_, Cs_3_Bi_2_I_9_, MA_3_Bi_2_I_9_, etc. were explored for X-ray detection, which generally exhibit less ion drift and larger resistivity [237, 250–252]. In addition to the single-pixel detector, the solution processability makes the printable flat-panel X-ray imager (FPXI) feasible [238]. Some practical methods like soft-sintering [11] and polymer–binder-assisted blade coating [253] were successfully demonstrated in FPXI. These innovative processing methods indeed accelerate the development of perovskite-based X-ray detectors and imagers. However, there remain several critical challenges. The high dark current is often observed in the perovskite detectors, which can quickly fill up the storage capacitance in the back panel prior to X-ray illumination and results in a poor dynamic range and poor signal-to-noise ratio (SNR) [246, 247]. To integrate with FPXI, sub-nA/cm^2^ dark current under working bias is generally required. The drift of the baseline dark current makes the situation even worse, which can be attributed to the ion migration in perovskite. Extensive efforts have been made to reduce the dark current very recently, including the construction of heterojunction of 3D/2D perovskite, the insertion of insulating polymer between the electrode and perovskite, and the separation of electronic and ionic transport pathways [253–256]. But most of the above methods simultaneously increase the resistivity, which in turn reduces the sensitivity. A balance needs to be achieved. In order to make the perovskite detector practicable, the current drift has to be addressed. This issue can be simply characterized by X-ray pulse-train measurement, and the stability under repeatable pulses can be taken as an important figure of merit when evaluating perovskite X-ray detectors. Just like many other perovskite-based optoelectronic devices, ion migration is a critical origin responsible for many undesirable characteristics of X-ray detectors.

### Challenges Ahead

Perovskites have been demonstrated as promising photodetection materials for high-efficiency photodetectors and imaging arrays due to their low cost, facile manufacturability and remarkable optoelectronic properties. Some figures of merit of perovskite photodetectors are comparable with that of commercial silicon and germanium counterparts [209], as shown in Table 5. To advance perovskite-based PDs and imaging arrays toward commercial applications, further exploration and efforts should be made to address following issues, including spectral response extension to longer wavelengths, the integration of pixel devices, flexibility, stability and competition from those mature technologies [202]. Notably, ion migration due to the ionic nature of perovskite could severely deteriorate the performance and stability of perovskite photodetectors [201], which demands further attention. Moreover, applications in novel fields like bioimaging, wearable devices and display put forward higher requirements for the flexibility and integration of perovskite-based photodetectors.

**Table 5:** Summary of performance of photodetectors with different perovskite materials

Photoactive materials	Responsivity (mA W^−1^)	Specific detectivity (Jones)	EQE (%)	t_rise_/t_fall_ (μs)	References
MAPbI_3-x_Cl_x_	–	8.00 $\times {10}^{13}$	80	0.18/0.16	[205]
FA_1-x_Cs_x_PbI_3_ /PdSe_2_	313	1.00 $\times {10}^{13}$	–	3.5/4	[9]
CsPbBr_3_	3150	3.94 $\times {10}^{12}$	–	8000/6500	[208]
MAPbCl_3_	46.9	1.20 $\times {10}^{10}$	–	0.024/0.062	[194]
FAPbI_3_	4500	-	900	0.0083/0.0075	[211]
MASnPbI_3_	200	~ 1 $\times {10}^{12}$	100	2.27	[219]
CsPbCl_3_	220	4.06 $\times {10}^{13}$	–	1.92/0.45	[8]
MAPbBr_3_	5040	5.37 $\times {10}^{12}$	1200	80/110	[204]

“–”: not available

## Perovskite Lasers

**Fig. 5:**
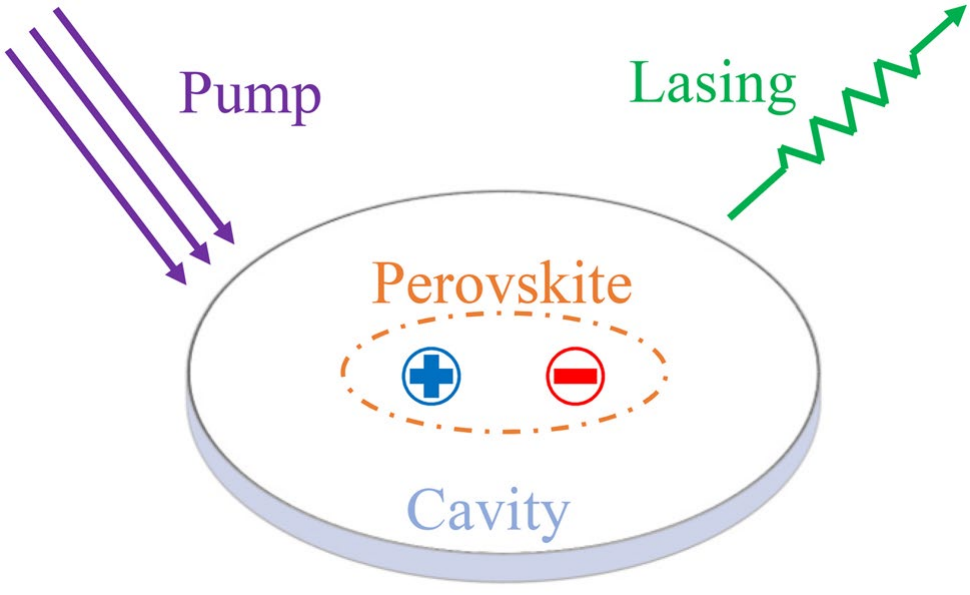
Schematic diagram of perovskite laser

**Fig. 6:**
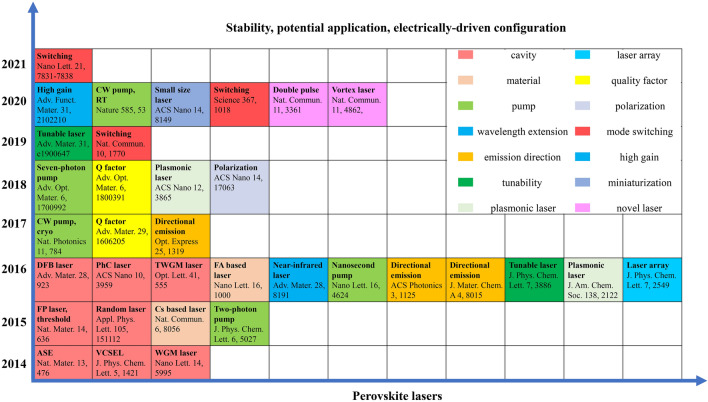
Progress timeline of perovskite lasers

Laser, an acronym for light amplification by stimulated emission of radiation, is generally composed of three key elements, specifically, gain medium, optical feedback resonator and pumping source. Perovskites are proved to be suitable materials to be used in laser devices, the schematic diagram of which is shown in Fig. 5. As direct-bandgap semiconductors, halide perovskites allow radiative recombination of photoinduced carriers, specifically, free electrons and holes or excitons, which depends on the exciton binding energy. Optical gain has been achieved via pulsed excitation at room temperature or even continuous-wave excitation under low temperature. Therefore, perovskite lasers have been realized in self-assembled perovskite micro- and nanoparticles or by integration with external optical cavities. Moreover, the patterning and integration techniques together with the rich diversity of perovskite compositions and morphologies promise huge potentials for perovskite lasers. In this section, we will first review the development history in the perovskite lasers briefly (Fig. 6) and then focus on the milestones and breakthroughs. Finally, we will discuss the challenges and opportunities for the perovskite lasers.

### Development History

Under pulsed laser excitation, Xing et al. observed amplified spontaneous emission (ASE) behavior in solution-processed 3D MAPbI_3_ perovskite thin film for the first time [257], indicating the intrinsic suitability of perovskites for coherent light emission. The net model gain, determined based on a variable stripe length method, has increased from initial 250 cm^−1^[257] to over 3000 cm^−1^ [258] in the solution-processed perovskite thin films. Moreover, the correlation between the gain regime and material bandgap has endowed perovskites with wide gain tunability spanning from visible to near-infrared range [259]. The optical feedback resonators are formed by natural structures of as-synthesized perovskites or by incorporating perovskites with external resonant cavities. At the early stage, the perovskite films exhibited poor crystal quality with rough and disordered surface. Random lasers have been implemented based on the mirrorless cavities, which are formed by multiple light scattering among the structural disorder [260]. Alternatively, vertical-cavity surface-emitting lasers (VCSELs) have also been realized by inserting perovskite films between gold mirror and distributed Bragg reflector [261]. Perovskite nanowires serve as gain media and resonant cavities simultaneously and afford axial Fabry–Perot (FP) lasing modes [262]. Whispering gallery (WG) mode lasers based on totally internal reflection at the lateral facets have been demonstrated in perovskite triangular, square and polygonal microplates [263]. Benefitting from the advancement in synthesis conditions and fabrication techniques [264], perovskite-based microspheres [265, 266], circular microdisks [267], microrings [268], microtubes [269] and microcapillary-assisted composite structures [270] have been prepared to achieve WG mode lasing. Interestingly, a transverse WG mode has been reported in the cross-sectional plane of perovskite microrods, pointing to the complexity and possible competition between FP and WG modes in individual microrods [271]. Besides, distributed feedback (DFB) lasers [272] and photonic crystal (PhC) lasers [273] have also been realized. For perovskites, population inversion could be obtained under pulsed optical pumping, evolving from femtosecond to nanosecond pulses [274]. With further development, continuous-wave (CW) pumped lasing actions have been realized in MHP [13, 275–277]. Note that the above-mentioned lasers are all pumped under external laser sources with a higher energy than the perovskite bandgap. In terms of the relatively large nonlinear effect of perovskite, two-photon and multiphoton (up to seven-photon) pumped lasers have been demonstrated [278, 279]. Moreover, perovskites as saturable absorbers have been used in Q-switched and mode-locked fiber lasers [280, 281].

### Current Progress for Perovskite Lasers with Different Metrics

Key metrics relating to lasing performance involve threshold, quality (Q) factor, emission direction, wavelength tunability, physical size, mode volume, polarization, modulation/switching, laser array and stability.

In 2015, Zhu et al. reported FP mode lasing in solution-processed perovskite nanowires with high crystal quality and demonstrated the lowest lasing threshold (~ 220 nJ cm^−2^) and the highest Q factor (~ 3600) at that time [262]. By virtue of better light confinement of WG mode, Zhang et al. achieved a high Q factor of ~ 6000 by using the single-crystal MAPbBr_3_ perovskite microdisks, which were prepared via COMS-compatible semiconductor manufacturing techniques [267]. Tang et al. synthesized all-inorganic perovskite microspheres via chemical vapor deposition method and realized up-converted WG mode lasing with the highest Q factor over 30,000 under two-photon pumping at low temperature [282].

Owing to the inherent emission characteristics of VCSELs, normal to the device surface directional emission with relatively small angular divergence can be expected [283]. By fabricating a specially designed cavity with slight modification from limacon shape on perovskite microplate, directional lasing emission due to the presence of unstable manifolds has been implemented by Zhang et al. [267]. Wang et al. synthesized perovskite-based waveguide connected microdisks via chemical engineering and demonstrated unidirectional lasing emission along the waveguide [284]. In this scheme, resonant modes mainly distributed in the square microdisk, whereas the waveguide functioned as leaky channel for the resonant modes. To reduce the propagation loss in the perovskite waveguide due to reabsorption, Cegielski et al. [285] integrated perovskite microring laser with silicon nitride waveguide to improve the outcoupling efficiency. Similarly, commercial optical fiber is also introduced to increase collection efficiency of perovskite microlasers through evanescently coupling with tapered fiber [286] or transferring perovskite microstructures onto the core of fiber [287].

Thanks to the rich chemical and dimensional versatility, the optical bandgap of perovskites can be easily tailored via controlling the composition and stoichiometric ratio. However, once synthesized, perovskite lasers show fixed and static emission wavelength. Based on the halide substitution, Zhang et al. [288] reported a post-synthetic method to tune the lasing wavelength from green to cyan. By selectively exposing MAPbBr_3_ perovskite microstructures to chloride in inductively coupled plasma machine, precise control on the wavelength shift can be realized by controlling the reaction time and heterostructure laser has also been demonstrated with simultaneous double-color emission by site-selective exposure. More importantly, the lasing threshold and output intensity could be well preserved during the whole reaction process. Yang et al. [289] demonstrated dynamic lasing wavelength tuning by applying compressive or tensile strain to the perovskite microwire on flexible substrate, where the shift was ascribed to the refractive index change of perovskite induced by piezoelectric polarization effect.

The physical size of perovskite lasers ranges from millimeter to micrometer scale. Tiguntseva et al. [290] reported single-mode lasing governed by Mie resonances from individual perovskite nanocubes as small as 310 nm, which is the most compact photonic nanolaser. To effectively scale down the physical size of lasers, metal-based cavities operating in the dielectric modes or plasmonic modes with much compressed mode volume have been proposed and experimentally demonstrated based on perovskites. Li et al. [291] embedded silver nanowires into the perovskite crystals and guided the photonic lasing mode from perovskite along the nanowire in the form of surface plasmons. Huang et al. demonstrated hybrid plasmonic nanolasers by transferring perovskite nanosheets onto gold substrate with an insulating spacer [292]. The plasmonic lasing mode showed transverse magnetic polarization with dominant electric field perpendicular to the substrate surface and obviously shortened lifetime above threshold compared with photonic lasing mode. Notably, plasmonic nanolaser amplifies surface plasmons rather than propagating photons, thus providing amplification of light localized at a scale smaller than the diffraction limit.

In general, the polarization characteristics of lasing emission depend on the resonant cavity and intrinsic merits of gain materials. For most of perovskite lasers, linear polarization is reported. Dai et al. [293] achieved circular polarization by integrating linearly polarized perovskite microlaser with geometric phase-based dielectric metalens. In principle, linear polarization can be decomposed into left-handed circular polarization (LCP) and right-handed circular polarization (RCP). Moreover, metalens is specially designed so that LCP incident light at the focal point can be collimated to RCP, whereas RCP diverged to LCP. Consequently, directional lasing emission with small angular divergence and chiral emission with dominant RCP have been realized simultaneously.

Photoluminescence (PL) intensity modulation of perovskites under external electric field has been widely investigated and the underlying mechanism has been ascribed to electric field-induced defect regulation and charge carrier transport [294–297]. By lifting the level degeneracy in neutral nanocrystals, Qin et al. [298] achieved electrical switching of ASE in perovskite nanocrystal films with over 50% threshold reduction under current injection. Concerns on the electrical modulation speed are raised considering that the response time of PL intensity change is at the scale of 100 ms. To deal with the modulation speed, Zhang et al. [299] demonstrated all-optical switching in individual perovskite microwires. Based on modal interaction via cross-gain saturation, reversible and ultrafast switching between two lasing modes could be implemented by varying the pump fluence. Taking 10-dB extinction ratio as a bench mark, the switching time could be identified, which is less than 100 ps.

The advancement in synthesis process and fabrication techniques for perovskite nanostructures has given rise to the development of perovskite laser array [264]. Typically, perovskite microcrystals are arranged in periodic distribution by splitting the perovskite precursor and confining the nucleation/growth sites. Alternatively, top-down nanofabrication technologies can also be leveraged to pattern monolithic perovskite crystals or films into periodic resonant structures. Under optical excitation, perovskite microlaser array forms. Inevitably, variations in shape and size occur among different unit cells due to the fabrication imperfection, leading to non-uniformity in lasing emission. Additionally, the size of unit cell ranges from several to tens of micrometers, which sets a limit to the integration density of laser array. Wang et al. [300] proposed a novel design and achieved high-density and uniform nanolaser array by tailoring the substrate. On the basis of the transverse WG mode [271], periodic leaky loss was introduced by transferring perovskite microwires onto silicon nanograting [301] and only the suspending parts in the air gap support lasing action. Benefitting from high crystal quality and uniform size distribution along the axial direction of perovskite microwire, negligible changes could be observed in different units of the laser array. In this configuration, a record integration density of 1250 laser units per millimeter has been demonstrated.

Perovskites are vulnerable to polar solvent, heat, humidity, light and energetic ions. To address the long-term stability issue, strategies including composition engineering and encapsulation are widely used. For example, cesium-based all-inorganic perovskites show better thermal- and photostability compared with the organic counterparts. Waterproof perovskite lasers have also been demonstrated by encapsulating perovskite with polymer, quartz microcapillary and hexagonal boron nitride [302–304].

### Novel Optical Phenomena

With the development of perovskite lasers, novel optical phenomena have been experimentally observed. In perovskite multi-quantum-well structure, Guo et al. demonstrated double-pulsed stimulated emission with pulse duration of 40 ps and interval of 70 ps [305]. Two-step carrier funneling process has been proposed to account for the phenomenon, during which vertical carrier funneling is faster than the lateral one. Huang et al. reported bound state in the continuum by patterning perovskite film with planar PhC structure [14]. In the far field, the intensity distributed in a donut shape with a dark core, which originated from the phase singularity at the beam axis. Moreover, the mode symmetry and far-field properties have been employed to realize all-optical switching, breaking the trade-off between low energy consumption and high modulation speed. Preliminary applications of perovskite lasers have been demonstrated in chemical gas sensing based on the organic stimuli-induced resonant wavelength shift [306]. In contrast to the utilization of Q factor or narrow linewidth of laser, Wang et al. exploited the ultrasmooth surfaces of single-crystal perovskite microplate in nanoparticle detection and high-resolution imaging [307, 308]. The scatters or nanostructures on the perovskite surface can convert evanescent wave to propagating wave, which is detectable in the far field.

### Challenges Ahead

Although recent years have witnessed the booming and substantial development in perovskite lasers, there is still plenty of room to improve: (1) Albeit large bandgap tunability has been achieved in perovskite lasers. The widely adopted composition engineering route, especially halide mixing, faces severe long-term stability issue, which originates from spontaneous or light-driven ion migration. (2) The toxicity of lead element should be addressed via developing tin-based or double metal halide perovskites with material properties suitable for lasing emission. (3) With the advent of CW pumped perovskite lasers, commercialization inspires the pursuit of electrically driven perovskite lasers. Similar to other photovoltaic and optoelectronic devices, up-scale manufacturing techniques should also be developed for perovskite lasers. (4) For perovskite lasers, the stability, especially photostability, determines the operation lifetime. Under high carrier or current injection, ion migration and heat accumulation accelerate the decomposition of perovskite. Hence, strategies on the suppression of ion migration and heat management should be further explored.

## Perovskite Neuromorphic Devices

### Fundamentals

Restricted by the von Neumann bottleneck, traditional computing systems that process information in time sequence usually suffer from large-space occupation and high energy consumption [309]. The emergence of neuromorphic electronics provides a more efficient working paradigm, inspired by that of the human brain and peripheral nervous system, which process information in parallel. To further emulate biological nervous system, artificial synapses (AS) and memristors emerge, which combine the functions of computation and memory in a single device to avoid Von Neumann bottleneck as caused by the frequent communication between computation and memory modules [310].

As the basic structural and functional units of a nervous system, synapses connect axons and dendrites to complete the transmission of neural signals with ultralow and event-driven energy consumption. Synaptic plasticity is achieved by adjusting synaptic weight [311]. When presynaptic spikes are applied to excite an AS, the conductivity of the device changes continuously. So, the emulation and control of plasticity can be realized by adjusting the form of presynaptic spikes, whose sensitivity and energy consumption are even comparable with biological level.

Memristor is the fourth basic circuit element except for resistors, capacitors and inductors, the concept of which was first proposed by Professor Chua in 1971 [312]. But it was not known by public until 2008 when Strukov et al. produced memristors experimentally for the first time [313]. The memristor is a nonlinear resistor with charge memory function, whose resistance is determined by the charge flowing through it. The resistance can be reversibly switched between high value and low value under certain applied voltage, which can be exploited to achieve “writing” and “erasing” processes in data storage [314].

Nowadays more and more materials have been utilized as the functional layer of neuromorphic devices, such as metal oxides, chalcogenides, metal nitrides, Ag/Si mixture, silicon oxides, organic semiconductor materials and 2D materials [315]. The silicon-based and metal oxides devices excel in durability and stability, but they suffer from complicated fabrication and relatively high switching voltage, rendering more power consumption. Chalcogenides, organic semiconductor materials and 2D materials have poor stability and complex preparation processes. Therefore, novel materials are highly desirable to improve device performance, such as perovskites and conductive polymer. Halide perovskites (HPs) enjoy the merits of low energy cost, solution processibility and excellent photoelectronic properties, like adjustable band gap, high quantum efficiency, long carrier lifetime, etc., rendering them suitable materials to be used in AS and memristors [16, 17].

### Current Progress

#### Perovskite-based Artificial Synapses

AS is a typical neuromorphic device emulating a biological synapse, which is designed to realize the processing and storage of spatiotemporal information from multifunctional sensing terminals. According to device architectures, AS can be classified into two-terminal devices with a “metal–functional layer–metal” sandwiched structure and synaptic transistors with similar structures as thin-film transistors. Compared to traditional silicon-based electronics, AS based on emerging materials give chance to brain-like computing and neuromorphic perception.

**Fig. 7:**
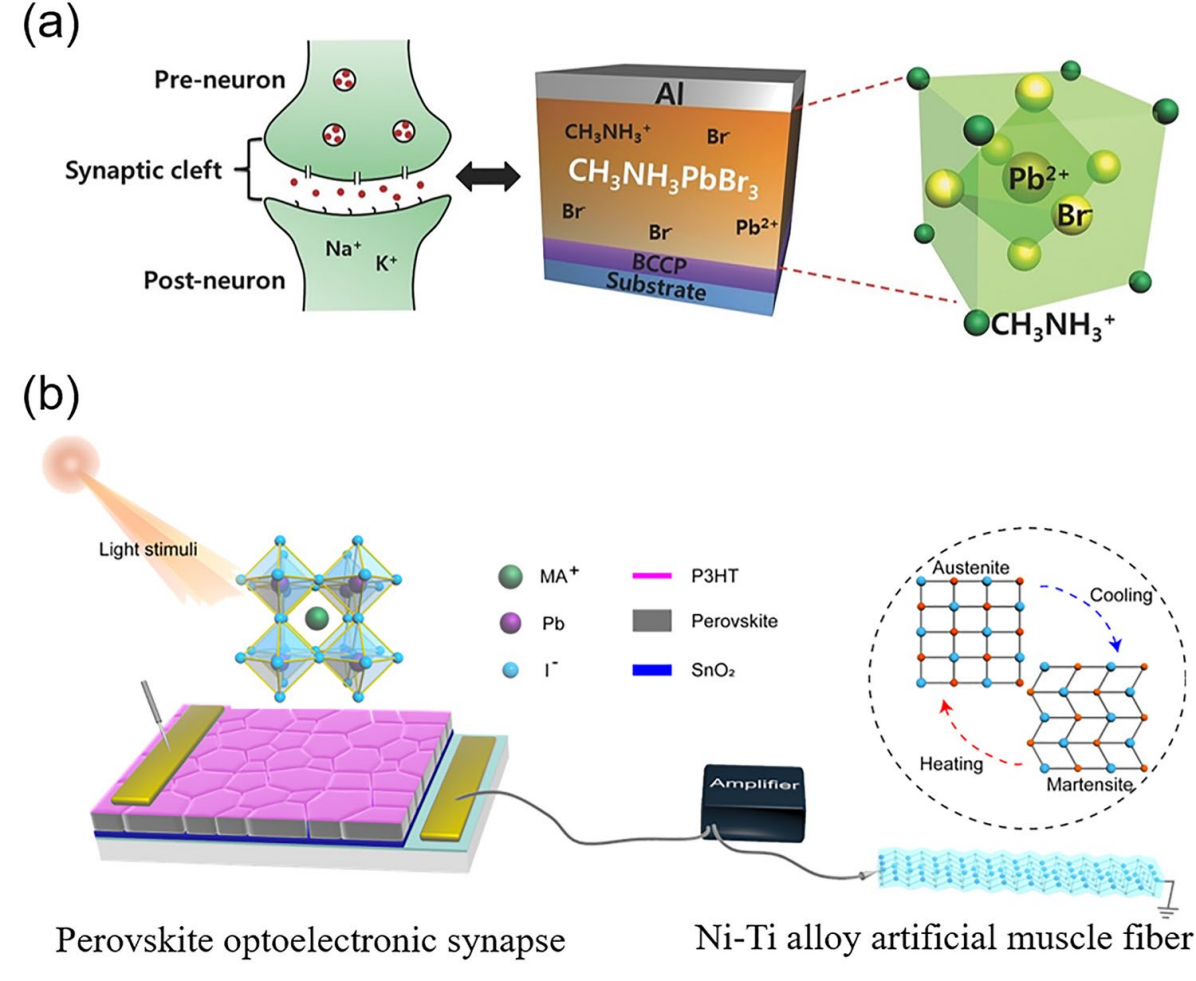
**a** Schematic demonstrations of CH_3_NH_3_PbBr_3_-based perovskite artificial synapse device. Reproduced with permission [316], Copyright 2016, John Wiley & Sons, Inc. **b** Schematics of an artificial visual nerve mimicking pupil reflex. Reproduced with permission [317], Copyright 2022, Elsevier

As one of the most excellent photoelectric conversion materials, organometal halide perovskites (OHPs) are widely used in photovoltaics, light-emitting diodes, transistors and photon detectors. OHPs sometimes suffer from ion migration, charge traps and ferroelectricity, leading to instability of the devices. However, these properties in turn benefit the consecutive conductance modulation of the OHP thin films, which can be used to emulate the tunable synaptic response of synapses. In 2016, Xu et al. [316] designed the first OHP-based AS, in which consecutive modulation of conductance of OHP thin film was achieved due to the electrical pulses-induced ion redistribution (Fig. 7a). Essential synaptic plasticity characteristics were emulated, including excitatory postsynaptic current (EPSC), paired-pulse facilitation (PPF), short-term potentiation (STP) and long-term potentiation (LTP).

In addition to neuromorphic computing, it is also important to construct a complete sensorimotor nervous system that integrates sensory and motor functions to synaptic units. Gong et al. [317] developed a p–i–n photoelectric synapse, which can achieve excitatory and inhibitory synaptic behaviors under light irradiation. By adjusting the external electric field on photogenerated current, the bidirectional synaptic weight can be enhanced and inhibited simultaneously, and thereby realized the emulation of excitatory and inhibitory responses of a nervous system. In addition, an artificial pupil reflex was constructed by combining the photoelectric synapse with nitinol artificial muscle fibers, which successfully emulated physiological behaviors of pupillary reflex (Fig. 7b).

In order to rival the performance of biological synapses, we need to fabricate AS with low energy consumption and high sensitivity to electrical pulses. Via a thickness-confined surfactant-assistant self-assembly method, Gong et al. synthesized a MAPbBr_3_ single-crystalline thin platelets (SCTPs) with controllable thickness and lateral size, whose surface roughness and trap density are lower than polycrystalline films. The perovskite SCTPs demonstrated good properties for optical responses and charge transport [318]. Then they constructed a perovskite SCTP-based AS with a lateral device architecture, where the anisotropic charge transfer in perovskite structures reduced energy consumption to approaching biological level (14.3 fJ/synaptic event).

0D perovskite quantum dots (QDs) not only have a stronger optical response but also can be operated in an optoelectrical dual mode in combination with another semiconductor layer. Wang et al. prepared CsPbBr_3_ QD-based photonic synapses with a three-terminal structure via thermal injection method. Separate modulation of photonic and electrical signals was realized, namely optically programmable and electrically erasable characteristics (photonic potentiation and electrical habituation). The synaptic weight could be regulated by multiwavelength light [319].

Huang et al. [320] designed synapses phototransistor based on a bipolar heterojunction of a non-fullerene acceptor material Y6 and 2D halide perovskite PEA_2_SnI_4_, endowing AS devices with the ability to learn in dual mode. The combination of Y6 with PEA_2_SnI_4_ broadened the absorption spectrum from visible to near infrared. Therefore, the heterojunction AS has a strong response to both visible and near-infrared light, which could be used as a multiwavelength acceptor and applied in color recognizable visual system.

#### Perovskite-based Memristors

HPs can be classified as ionic semiconductors and applied in memristors. In 2014, Xiao et al. discovered the memristive effect in perovskite with “ITO/PEDOT:PSS/MAPbI_3_/Au” structure, which enlightened the development of HP memristors. In 2015, Yoo et al. fabricated the first HP memristor with “FTO/MAPbI_3−x_Cl_x_/Au” structure, showing bipolar resistive switching (RS) behavior due to the ion migration [321]. When a positive voltage was swept from 0 to 1 V, the resistance switched from high-resistance state (HRS) to low-resistance state (LRS), corresponding to a set process. The resistance switched from LRS to HRS at − 0.6 V, corresponding to a reset process.

**Fig. 8:**
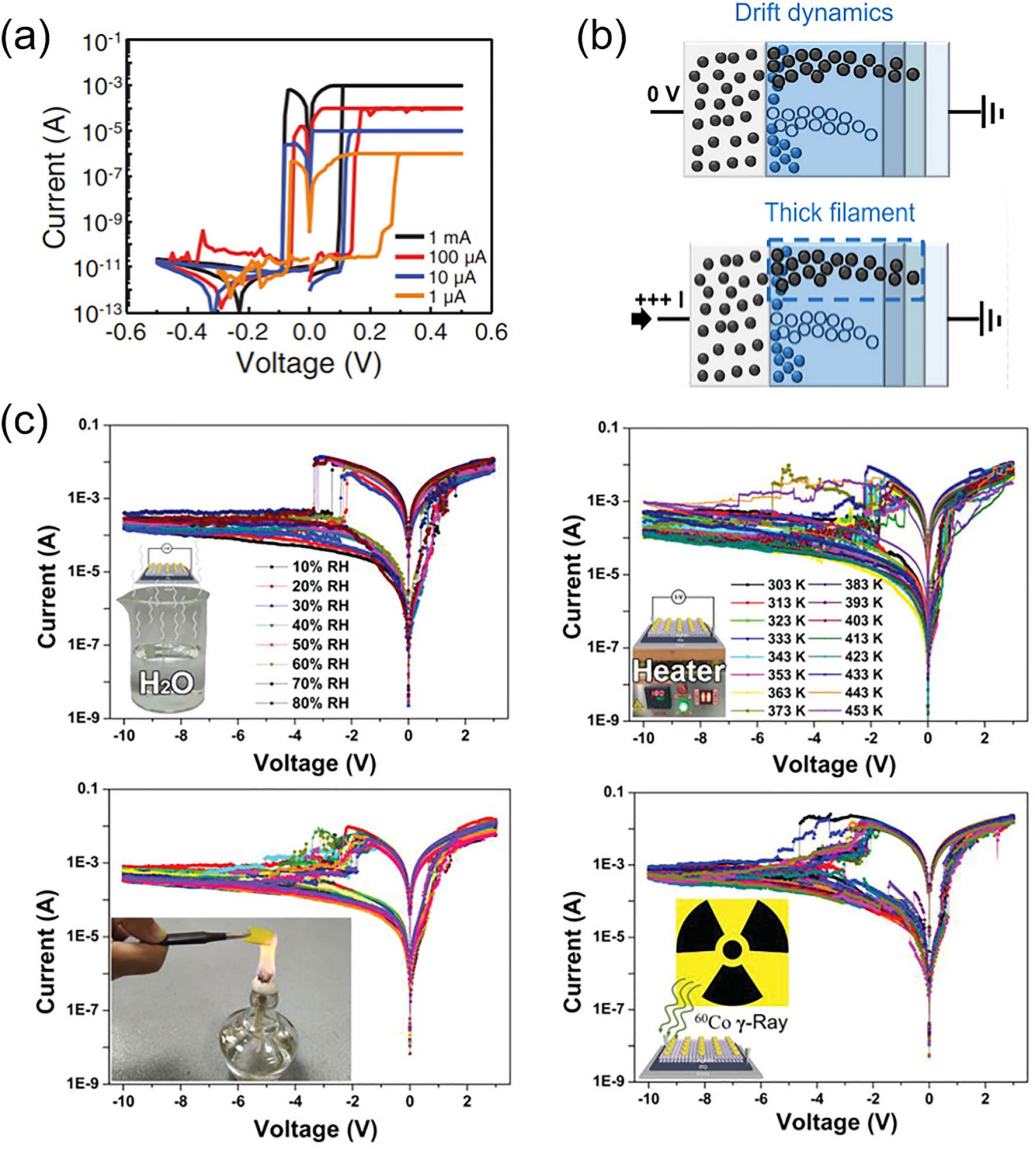
**a** Multilevel switching in *I–V* curves under the four different current compliance for Au/PMMA/dual-phase HP/Pt RS devices. Reproduced with permission [322], Copyright 2020, John Wiley & Sons, Inc. **b** Device structure for nonvolatile memristor with drift switching mechanism. Reproduced with permission [325], Copyright 2022, Springer Nature. **c** *I–V* characteristics of Cs_2_AgBiBr_6_-based memristor device in different harsh environments. Reproduced with permission [326], Copyright 2019, John Wiley & Sons, Inc

HP materials could be applied in nonvolatile resistive random-access memories (RRAM) due to the RS ability enabled by fast ion migration. High ON/OFF ratio is desirable, which can not only yield multilevel data storage but also improve the reliability of the device. A dual-phase AgBi_2_I_7_-Cs_3_Bi_2_I_9_-based memristor showed filamentary RS behavior and multilevel storage characteristic because of the high ON/OFF ratio (> 10^7^) (Fig. 8a) [322]. To date, the highest ON/OFF ratio for HP-based memristors has reached as high as 10^9^ [323]. Besides, fast switching speed is highly required for data processing in artificial synapses devices. Park’s group reported a dimer-Cs_3_Sb_2_I_9_-based memristor with fast switching speed of 20 ns due to the low vacancy–migration barrier [15]. The excellent light absorption characteristics of HP materials can be harnessed to suppress the conductive filaments (CF) overgrowth induced by the current overshoot in the electroforming process. In memristor devices with a “FTO/MAPbI_3_/Au” structure, the introduction of light irradiation in the initial electroforming process can promote the migration of iodide ions and improve the conductivity, which synergistically restrain the unwanted overgrowth of CF [324]. The progress of memristors in each structure is overviewed and the detailed information is summarized in Table 6.

**Table 6:** Performance of perovskite memristors with different structures

Structure	On/off ratio	Endurance (cycles)	Retention (s)	Environmental stability (days)	References
FTO/CH_3_NH_3_PbI_3-x_Cl_x_/Au	–	100	10^4^	14	[321]
Si/SiO_2_/ Ti/Pt/AgBi_2_I_7_-Cs_3_Bi_2_I_9_/PMMA/Au	10^7^	10^3^	5 × 10^4^	30	[322]
Si/SiO_2_/ Ti/Pt/(PEA)_2_Cs_3_Pb_4_I_13_/Ag	10^9^	200	2000	14	[323]
ITO/Cs_3_Sb_2_I_9_/Au	10^2^	500	5000	–	[15]
FTO/CH_3_NH_3_PbI_3_/Al	10	500	–	–	[324]
ITO/PEDOT:PSS/TPD/CsPbBr_3_/Ag	10^3^	500	10^4^	–	[325]
ITO/Cs_2_AgBiBr_6_/Au	10	10^3^	10^5^	100	[326]
ITO/CsBi_3_I_10_/Al	10^3^	150	10^4^	60	[327]

“–”: not available

The RS behavior of perovskite is mainly induced by ion migration, which can be divided into filament or band bending-type in terms of the switching mechanisms. For filament-type devices, RS behavior was induced by the formation and rupture of CF under the electric field, which was enabled by the mobile defects or ions in perovskites (Fig. 8b) [325]. For band bending-type devices, the ion migration reduces the Schottky barrier at the interface between active layer and electrode, which facilitates the charge transport and charge capture by traps at the interface. This leads to the resistance switching. It should be noted that the HP ionic characteristic is a double-edged sword. The ion migration can enable high performance and weaken the stability of memristors at the meantime. Lu et al. reported the first double perovskite memristors with a sandwich structure of “ITO/Cs_2_AgBiBr_6_/Au” to enhance the device stability, which can perform reproducible and reliable memristive behavior in harsh environments (Fig. 8c) [326]. Sun et al. used CsI-rich precursor solution to fabricated CsBi_3_I_10_-based stable RS devices, which showed good stability after over 2 month storage in an ambient (60% relative humidity) environment [327].

### Challenges Ahead

Since the first study of HP-based neuromorphic device, great progress has been made. The structure and function of the AS devices have been greatly optimized, which even showed comparable performance to the biological level in some figures of merit and plenty of applications in various areas, such as health monitoring, image recognition and artificial nerve construction. Perovskite-based memristors show great potential to be applied in RRAM and logic computing due to their unique properties such as multilevel resistance, high ON/OFF ratio and tunable composition. However, obstacles still exist to achieve mass production and commercial application, such as the poor stability of perovskite under external stimuli and lead toxicity. For AS and memristors, it is expected to be a potential candidate for human electronics, but bioelectronic compatibility, flexibility, environmental stability, productivity and so on affect its speed of development. In addition, the mechanism of the perovskite-based neuromorphic device is not fully explained because of the complex ion movement process, which demands further exploration to refine the design of future neuromorphic devices not only in functional materials but also structure. Still, it is undeniable that perovskites have shown fascinating potential in neuromorphic computing devices and is hoped to take a place in brain-like computing and artificial nervous system.

## Pressure-induced Emission

### Fundamentals

Low-dimensional halide perovskites (LDHPs) are considered as promising candidates for single-component white light emission benefiting from their broadband emission spanning almost across the whole visible spectrum [328]. The novel features of LDHP are mainly attributed to the soft lattice and strong exciton–phonon coupling, which give rise to the formation of self-trapped excitons (STEs). To date, it is increasingly challenging to obtain highly efficient STE emission in LDHP through conventional strategies like surface ligand passivation and shell coating. Actually, the distortion of halide octahedra would greatly affect the physical and chemical properties of the targeted LDHP. Novel strategies to improve STE emission efficiency are highly desirable. High pressure, as a thermodynamic extreme condition and a “clean” external stimulus, can effectively modulate the distortion degree of halide octahedra without altering chemical compositions. Accordingly, high pressure is expected to exert positive effect on STE emission in LDHP [329].

**Fig. 9:**
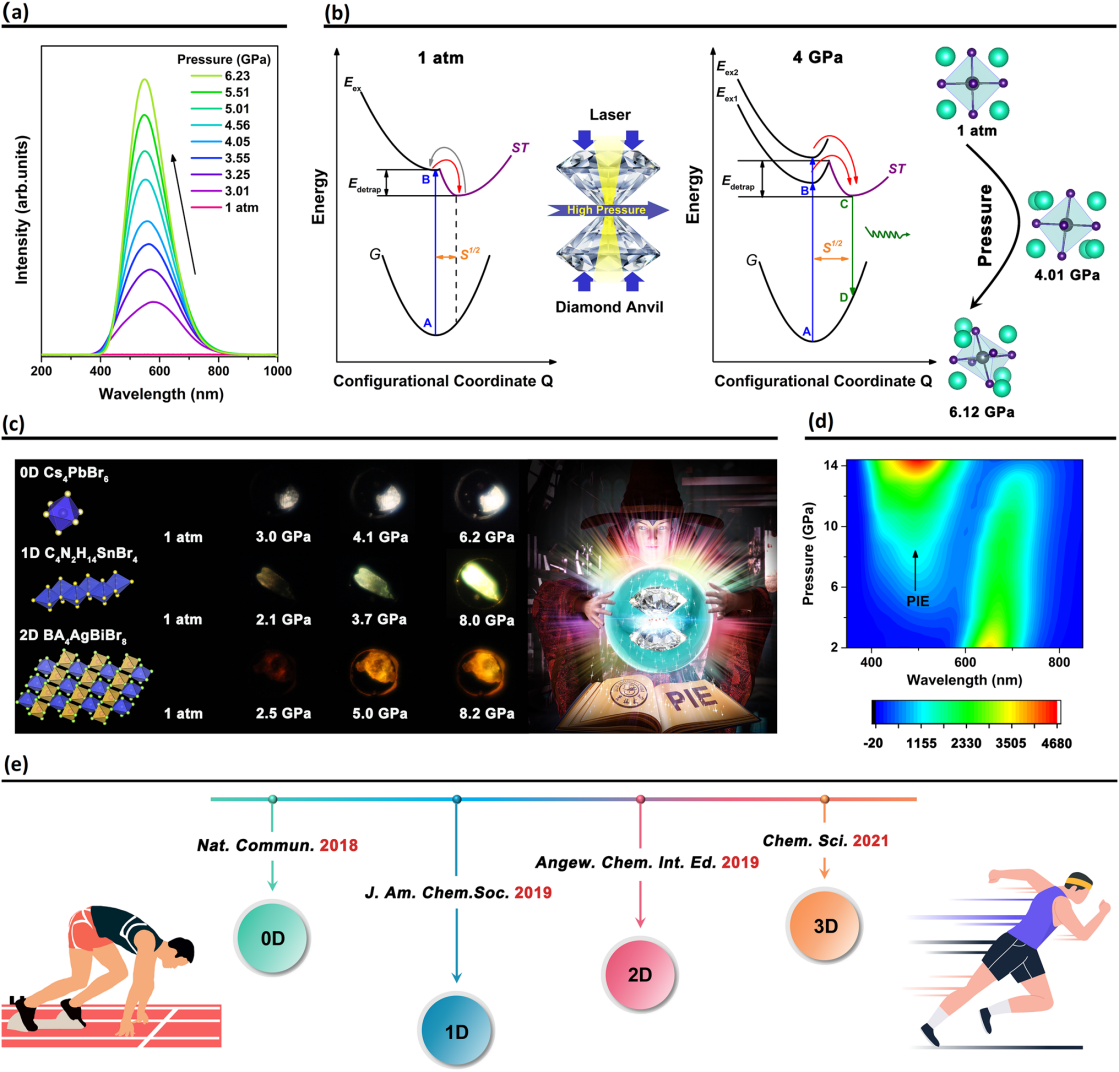
Advances of PIE from concept to applications **a** PL spectra of Cs_4_PbBr_6_ NCs under high pressure. **b** Illustration of PIE mechanism associated with exciton self-trapping in Cs_4_PbBr_6_ NCs. Reproduced with permission from Ma et al. [330], Copyright 2018, Nature Publishing Group. **c** Summary about PIE images of 0D Cs_4_PbBr_6_, 1D C_4_N_2_H_14_SnBr_4_ and 2D (BA)_4_AgBiBr_8_. Reproduced with permission from Ma et al. [330], Copyright 2018, Nature Publishing Group, Shi et al. [331], Copyright, 2019, American Chemical Society, and Fang et al. [332], Copyright 2019, Wiley–VCH. **d** 2D projection of PIE for Mn^2+^ doping CsPbBr_3_ NCs. Reproduced with permission from Shi et al. [333], Copyright 2021, Royal Society of Chemistry. **e** Progress timeline of PIE from inception to development across 0D to 3D halide perovskites

Cs_4_PbBr_6_, as a prototypical zero-dimensional (0D) perovskite, contains an array of isolated [PbBr_6_]^4−^ octahedra separated by Cs^+^ ions. Although possessing the lowest electronic dimensionality, Cs_4_PbBr_6_ nanocrystals (NCs) failed to exhibit any emission under ambient conditions, which largely limit their practical optoelectronic applications. In this regard, high pressure was invoked to identify the structure–property relationship and improve the luminescent properties of dense Cs_4_PbBr_6_ NCs [330]. As shown in Fig. 9a, when the applied pressure exceeds 3.0 GPa, the initially non-emissive Cs_4_PbBr_6_ NCs exhibit an exotic broad emission, accompanied by a phase transition from rhombohedral to monoclinic. The underlying mechanism was that the high-pressure-yielded octahedral distortion promotes the wave function overlap between ground states and excited states, thus elevating transition dipole moment and oscillator strength (Fig. 9b). Simultaneously, the electron–phonon coupling strength relevant to the Huang–Rhys factor is strengthened upon compression, which enhances the STE binding energy and prevents the reverse exciton transition from self-trapped states to bound states. Both the two factors as mentioned above jointly result in the newly emerging emission. New concept of pressure-induced emission (PIE) was thus proposed whereby a lightless material experiences abnormal emission upon compression.

### Current Progress of Perovskite PIE

Furthermore, PIE was subsequently corroborated in compressed one-dimensional (1D) halide perovskite C_4_N_2_H_14_SnBr_4_ and compressed two-dimensional (2D) halide perovskite BA_4_AgBiBr_8_, respectively (Fig. 9c) [331, 332]. Likewise, STE emission usually occurs in LDHP, whose relatively weak structural connectivity largely depresses the conductivity. Rendering traditional three-dimensional (3D) perovskites to create competitive STE emission is highly desirable to achieve stable white light devices. It is well established that regulating the octahedral distortion of halide perovskites significantly affects the trapping/detrapping process of free excitons, thus facilitating the realization of PIE. Doping engineering with mismatched dopants could lead to the occurrence of localized carriers. Through combining pressure processing, Shi et al. have recently achieved an intriguing PIE in a typical 3D perovskite, Mn-doped CsPbBr_3_ NCs, accompanied by high-quality white light emission with chromaticity coordinates of (0.330, 0.325) (Fig. 9d) [333]. This white light emission was because that high pressure could further induce the octahedral distortion of Mn-doped CsPbBr_3_ NCs to accommodate the STEs. The progress timeline of PIE from 0 to 3D is depicted in Fig. 9e, summarizing the milestone works of perovskites regarding PIE concept and applications.

The discovery of PIE further promotes the breakthrough of pressure-induced emission enhancement (PIEE). Recently, Lü et al. have identified the optimal relationship between PIEE and octahedral distortion involving the Huang–Rhys factor in halide perovskites and maximized photoluminescence (> 20 times) by regulating off-centering distortion [334]. Moreover, PIEE was also achieved in C_4_N_2_H_14_PbBr_4_ with 1D configuration [335, 336]. The quantitative method of PLQY under in situ gigapascal pressure was built and a dramatic PLQY of 90% at 2.8 GPa was obtained. In addition, metal halides, a class of perovskite variants that contain other polyhedral units, have recently attracted enormous attention. Notably, the PIEE could also be realized in these perovskite derivatives, like CsCu_2_I_3_ with CuCl_4_^3+^ tetrahedral units [337]. The apparent structural distortions of both inter- and intra-tetrahedra were responsible for the significant PIEE.

### Challenges Ahead

Nowadays, research of PIE has entered a new era. The PIE has the potential to resolve some scientific disputes, such as the long-running conventional cognition about the origin of green emission in Cs_4_PbBr_6_ NCs and extremely weak narrow blue emission in indium-based double perovskites [338–340]. Although significant progress has been made recently in PIE field, there are still great challenges. Firstly, exploring new methods, such as high-pressure chemical reaction, to quench the high-efficiency emission to the ambient conditions is of great significance to harvest bright materials. A strategy was recently proposed through building steric hindrance by introducing complexly configurational organic molecules to increase the potential barrier and thus preventing the high-pressure metastable state from returning to the initial stable state after pressure release [18, 19]. Secondly, advanced calculations, such as CALYPSO and machine learning, and in situ high-pressure technologies including neutron scattering, pair distribution function as well as time–space resolved transient spectra, should be cooperatively carried out to offer clear insights into the underlying mechanisms of PIE. Last but not least, PIE is expected to step toward not only fluorescence, but also delayed fluorescence, phosphorescence and chiral luminescence, thus significantly promoting the applications of anti-counterfeiting, information storage, sensing and display.

## Conclusion and Outlook

To sum up, we systematically summarized the recent advances and outlined the future challenges for perovskite materials in applications of solar cells, LEDs, photodetectors, lasers, artificial synapses, memristors and pressure-induced emission. Up to now, significant progress has been made in perovskite-based materials and devices. However, there is still plenty of room for further improvement of performance for each application and it is still far away from commercialization. Challenges and opportunities coexist. As a result, a comprehensive knowledge of the current progress, research hot spots and future directions is of vital importance.

Among all applications, perovskite solar cell is the most promising optoelectronic device toward commercialization, since the efficiency has been comparable to that of crystal Si solar cells. But there is still a gap between the state-of-the-art level and S-Q limit. Challenges of upscaling, stability issue and lead toxicity retard its commercialization process. PeLEDs have shown great potential to be used as light sources and displays due to the rapidly growing EQE of red, green and NIR LEDs during these years. But obstacles of inferior performance of blue LEDs, poor efficiency of white PeLEDs and severe efficiency roll-off strictly limit its further development. Besides, perovskites have been recognized as potential photodetection materials for photodetectors and imaging arrays. Some figures of merit of perovskite photodetectors even rival that of commercial silicon and germanium counterparts, but it is still challenging to compete with them with respect to overall performance. As for lasers, although perovskite lasers have made considerable achievements, the intrinsic instability nature of perovskite materials remains the major problem. Novel applications involving artificial synapses devices, memristors and pressure-induced emission also have made encouraging progress. Notably, perovskite neuromorphic devices including artificial synapses and memristors offer opportunities for perovskite materials to keep pace with the latest information technology revolution. The mechanisms behind still need deep exploration.

With more profound insights and further developments, we believe that some perovskite-based devices will meet the requirements for practical applications and step into commercialization in the future.
